# Using vulnerability assessment to characterize coastal protection benefits provided by estuarine habitats of a dynamic intracoastal waterway

**DOI:** 10.7717/peerj.16738

**Published:** 2024-02-19

**Authors:** Gregory M. Verutes, Philip F. Yang, Scott F. Eastman, Cheryl L. Doughty, Therese E. Adgie, Kaitlyn Dietz, Nicole G. Dix, Allix North, Gregory Guannel, Samantha K. Chapman

**Affiliations:** 1 Blue Forest Conservation, Sacramento, CA, United States; 2 Center for Biodiversity and Ecosystem Stewardship and Department of Biology, Villanova University, Villanova, PA, United States; 3 Guana Tolomato Matanzas National Estuarine Research Reserve, Ponte Vedra Beach, FL, United States; 4 Department of Geography, University of California, Los Angeles, Los Angeles, CA, United States; 5 Caribbean Green Technology Center, University of the Virgin Islands, St. Thomas, Virgin Islands, United States

**Keywords:** Coastal vulnerability, CVI, Natural capital, Ecosystem services, NERRS, Florida

## Abstract

The existence of coastal ecosystems depends on their ability to gain sediment and keep pace with sea level rise. Similar to other coastal areas, Northeast Florida (United States) is experiencing rapid population growth, climate change, and shifting wetland communities. Rising seas and more severe storms, coupled with the intensification of human activities, can modify the biophysical environment, thereby increasing coastal exposure to storm-induced erosion and inundation. Using the Guana Tolomato Matanzas National Estuarine Research Reserve as a case study, we analyzed the distribution of coastal protection services–expressly, wave attenuation and sediment control–provided by estuarine habitats inside a dynamic Intracoastal waterway. We explored six coastal variables that contribute to coastal flooding and erosion–(a) relief, (b) geomorphology, (c) estuarine habitats, (d) wind exposure, (e) boat wake energy, and (f) storm surge potential–to assess physical exposure to coastal hazards. The highest levels of coastal exposure were found in the north and south sections of the Reserve (9% and 14%, respectively) compared to only 4% in the central, with exposure in the south driven by low wetland elevation, high surge potential, and shorelines composed of less stable sandy and muddy substrate. The most vulnerable areas of the central Reserve and main channel of the Intracoastal waterway were exposed to boat wakes from larger vessels frequently traveling at medium speeds (10–20 knots) and had shoreline segments oriented towards the prevailing winds (north-northeast). To guide management for the recently expanded Reserve into vulnerable areas near the City of Saint Augustine, we evaluated six sites of concern where the current distribution of estuarine habitats (mangroves, salt marshes, and oyster beds) likely play the greatest role in natural protection. Spatially explicit outputs also identified potential elevation maintenance strategies such as living shorelines, landform modification, and mangrove establishment for providing coastal risk-reduction and other ecosystem-service co-benefits. Salt marshes and mangroves in two sites of the central section (N-312 and S-312) were found to protect more than a one-quarter of their cross-shore length (27% and 73%, respectively) from transitioning to the highest exposure category. Proposed interventions for mangrove establishment and living shorelines could help maintain elevation in these sites of concern. This work sets the stage for additional research, education, and outreach about where mangroves, salt marshes, and oyster beds are most likely to reduce risk to wetland communities in the region.

## Introduction

Coastal and estuarine habitats provide myriad benefits to nature and people, known as ecosystem services. They are estimated worldwide to be worth US$18.0 trillion/year (Costanza et al., 2014). These ecosystem benefits originate as natural capital stocks, providing coastal communities with provisioning (*e.g*., fisheries, aquaculture production), regulating (*e.g*., shoreline protection and flood control), cultural (*e.g*., wildlife viewing and educational opportunities) and supporting services (*e.g*., filtration of pollution and habitat for aquatic and terrestrial species) (Millennium Ecosystem Assessment Program (MEA), 2005). Mangrove ecosystem services alone, not including carbon sequestration, were valued at 193,845 US$ per hectare of an intact ecosystem as a global average (De Groot et al., 2012) and may be undervalued due to a lack of accounting for all ecosystem benefits (Barbier, 2016; Himes-Cornell, Grose & Pendleton, 2018). To assess ecosystem-service value at the regional or local scale requires a detailed understanding of multiple factors, which can vary by site and over space and time (Azevedo, Duarte & Bordalo, 2008; Rosenthal et al., 2015; Ruckelshaus et al., 2015).

The ecosystem benefits provided by estuarine habitats in the southeastern United States are many and varied (Table 1; see Barbier, 2015; Menéndez et al., 2020). Mangroves, salt marshes, and oyster beds filter pollutants and improve the quality of nearshore waters, supporting the livelihoods of coastal-dependent communities, and are a source of revenue from ecotourism (Coen et al., 2007; Batker et al., 2010; Barbier, 2015). Marshes are biologically diverse habitats that continuously accumulate sediment and have adapted to fresh and saltwater (Craft, 2007). They filter nutrients and sediments of flowing water, protect coastlines against wave damage and erosion, mitigate flooding by holding excess storm waters, and regulate climate (Sifleet, Pendleton & Murray, 2011; Shepard, Crain & Beck, 2011; Georgiou & Turner, 2012). Historically, native oyster beds could filter entire estuaries (Zu Ermgassen et al., 2012) and in some less degraded estuaries, oyster populations still provide significant filtration services (60%; Gray et al., 2021). Estuarine habitats also serve as natural buffers from hazards, reducing the need for costly investments in seawalls, riprap, bulkheads, and other types of ‘hard’ shore protection (Shepard, Crain & Beck, 2011; Barbier, 2015). These habitats have been shown to reduce wave heights by 80–90% (Barbier et al., 2013; Doughty et al., 2017). Marshes can be more effective at shoreline protection than their constructed counterparts, even during hurricanes (Gittman et al., 2014). Further, the combination of restored oyster structures with constructed breakwalls is more effective at mitigating shoreline erosion than hard shore protection alone (Safak et al., 2020a).

**Table 1:** Summary of estuarine habitats in GTMNERR known to attenuate wave energy, their potential in terms of coastal protection and ecosystem-service co-benefits, and regional threats. Italics indicate correspondence between each threat and the provided photos.

Habitat name	Wave attenuation potential (zone of influence)	Ecosystem-service cobenefits	Threats to habitats
Mangrove forests 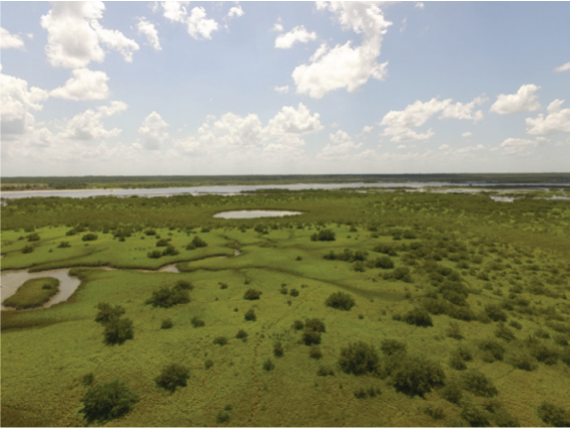	Highest (500 m)	Fisheries, blue carbon, water purification, habitat for flora and fauna	*Hardened shoreline*, dredging of channels, sea level rise, freeze events, boat wake exposure 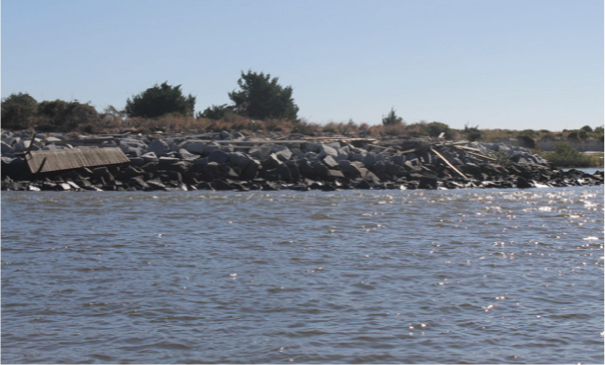
Salt marshes 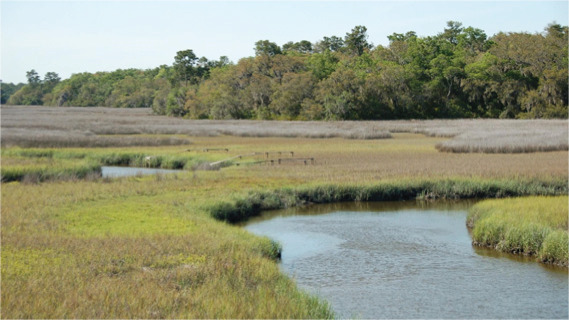	Medium (250 m)	Blue carbon, recreation, habitat for flora and fauna, fisheries, aesthetics	Coastal development, *mangrove encroachment*, sea level rise, marsh die backs, *boat wake exposure*, dredging 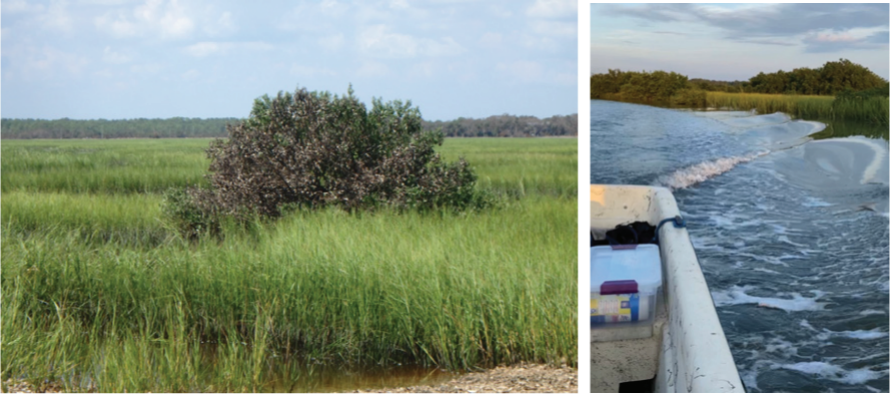
Oyster beds 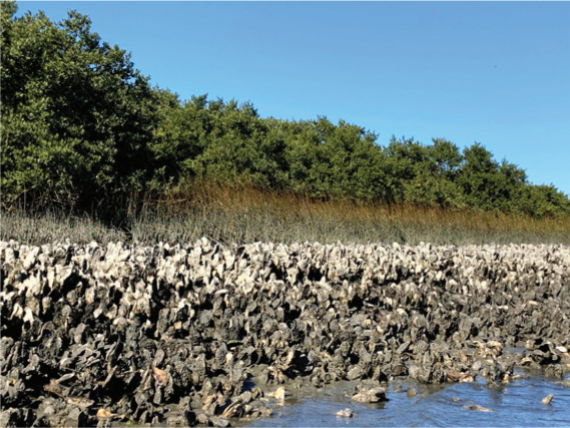	Medium (100 m)	Water purification, fisheries, habitat for flora and fauna, commercial and recreational harvest, blue carbon	Boat wake exposure, dredging of intracoastal waterway, unsustainable harvest, degradation of food quality *via* water pollution, increased water levels due to *sea level rise and storms* 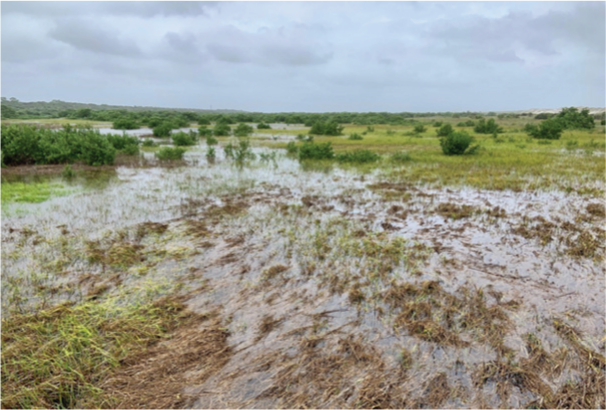

In contrast to biogenic habitats, seawalls and revetments are susceptible to overtopping (*i.e*., water rising over the top of the barrier) and increased wave reflection (Currin, Delano & Valdes-Weaver, 2008; Gittman et al., 2016). Consequently, engineered or ‘grey’ solutions can erode ecosystem integrity and reduce ecosystem service capacity overall (Bilkovic & Roggero, 2008; Long et al., 2011; Patrick et al., 2014). Coastal wetlands are increasingly under pressure from sea level rise and conversion for agricultural use or coastal development, mainly through dredging, filling, draining, and the construction of roads (Ellison & Farnsworth, 1996; Kennish, 2001). Further, estuarine ecosystems are changing dramatically in some ecotonal regions due to mangrove encroachment into former salt marshes due to climate change and other anthropogenic pressures (Cavanaugh et al., 2014; Doughty et al., 2016; Kelleway et al., 2017). The literature suggests a direct relationship between waves generated by boats, suspending sediment, and nearshore turbidity—all factors that alter estuarine ecosystem structure and function (Fonseca & Malhotra, 2012; Forlini et al., 2021; Safak, Angelini & Sheremet, 2021). Kennish (2001) cites boat wakes as a driver of bank erosion in coastal salt marshes, and these nearshore waves from boat activities also impact oysters (Grizzle, Adams & Walters, 2002). Approximately 85% of oyster beds have been lost worldwide (Beck et al., 2011), and dead intertidal reefs can form shell rakes that further contribute to coastal erosion (Garvis, Sacks & Walters, 2015).

When flooding leads to direct and indirect ecological damage to an ecosystem or causes damage to man-made structures, it is deemed a hazard. Certain regions can be more vulnerable to flooding than others (*e.g*., low-elevation human communities in the floodplain), and it is important to understand where and when severe events will occur (Few, 2003; Green, 2004). Flooding also negatively impacts coastal habitats (*e.g*., wetlands and marshes) when the duration of the event causes primary productivity to decrease, limiting mineral sedimentation and sediment organic matter accretion to maintain elevation (Morris et al., 2002; Morris, Sundberg & Hopkinson, 2013; Kirwan & Guntenspergen, 2015; Stagg et al., 2020). Storm surge floods can cause significant damage to life and property (Ebersole et al., 2010) and many studies have characterized the vulnerability of structures (Paleo-Torres et al., 2020; Chao, Ghansah & Grant, 2021) and populations (Wang & Yarnal, 2012; Hung, Wang & Yarnal, 2016) to rising water levels and flooding in Florida.

Florida’s coastal residents rely on estuarine ecosystems for livelihood and life-giving services (Price, 2005; Doughty et al., 2017; Narayan et al., 2019). Yet, paradoxically, the activities that make these benefits possible pose substantial risks. A 2013 United States (US) coastal vulnerability assessment identified the state of Florida as home to the most exposed property (total value in US$) and a disproportionately high elderly population, with increasing vulnerability projected across most of the Atlantic coast under four future sea-level rise scenarios for the year 2100 (Arkema et al., 2013). Threats from hazards to Florida’s coastal zone were front and center in October 2016 when Hurricane Matthew delivered 100+ mph winds to the First Coast of Florida (five northeastern counties along the Atlantic coast). It was the strongest storm to reach the area since 1898, with $149.4M in damages to St. Johns County alone and more than 119,000 flood insurance claims filed statewide (Florida Office of Insurance Regulation, 2021). Hurricane Matthew’s storm surge inundation impacted the St. Augustine area with flood levels of 6 to 7 feet above ground level (National Oceanic and Atmospheric Administration (NOAA), 2017). Since then, Hurricanes Irma (80 billion US$ in total cost), Ian, Nicole, and several nor’easters, combined with king tide and sea level rise, have repeatedly affected coastal infrastructure. Due to an increase in regional hazards, Florida’s environmental agencies and reserve systems monitor and communicate these social, economic, and ecological impacts to improve the decision-making ability of resource managers (Florida Department of Environmental Protection (FDEP), 2021; National Estuarine Research Reserve System (NERRS) Science Collaborative, 2022).

As human activities and climate change impacts intensify in coastal communities, nature-based solutions such as habitat conservation and restoration can bolster resilience (Pontee et al., 2016; Arkema et al., 2017) by reducing the exposure and vulnerability of ecosystems (Reiblich et al., 2019; IPCC, 2022). Current techniques for coastal vulnerability assessment are based on numerically ranked variables that include parameters to evaluate the physical exposure of the coastal zone to hazards (*e.g*., Gornitz, 1990; Palmer et al., 2011; Koroglu et al., 2019). Recent approaches for assessing coastal vulnerability in Florida use Geographic Information Systems (GIS) to identify suitable areas for habitat protection and restoration (Frazier, Wood & Yarnal, 2009; The Nature Conservancy (TNC), 2015; Calil & Newkirk, 2017; Narayan et al., 2019). However, these regional assessments may not capture salient drivers of exposure (*e.g*., boat wakes and the presence of hard structures) nor near-term changes in habitat quality and distribution. In addition to regional variation, the role of natural habitats in coastal protection varies locally by habitat type, distance to the coast, depth, and other factors (Guannel et al., 2016; Ruckelshaus et al., 2016). The magnitude of restoration possibilities combined with diverse coastal habitats necessitates understanding drivers of coastal vulnerability on a local scale to identify and apply the appropriate coastal restoration strategy.

Here, we describe the results of the first vulnerability assessment to our knowledge that estimates relative exposure to hazards inside a National Estuarine Research Reserve (USA). We assembled ranked information for six variables characterizing the biological and physical environment: (a) relief, (b) geomorphology, (c) estuarine habitats, (d) wind exposure, (e) boat wake energy, and (f) storm surge potential. Two variables (c and e) were derived using novel mapping and participatory techniques to account for dynamic ecotonal change (habitats) and fill knowledge gaps in smaller channels (boat wakes), providing more detailed data on the coastal vulnerability of adjacent sites. In addition, we present the results from a spatial prioritization of coastal areas with the greatest protection or enhancement need. Our findings identified (1) areas that are most exposed to wind and waves throughout and (2) where habitats play the greatest role in risk reduction (*e.g*., wave attenuation and sediment control) from coastal hazards. We conclude by discussing the next steps for pursuing wetland elevation maintenance strategies in the Reserve and follow-up initiatives of our research collaborative.

## Materials and Methods

### Study area

**Figure 1:**
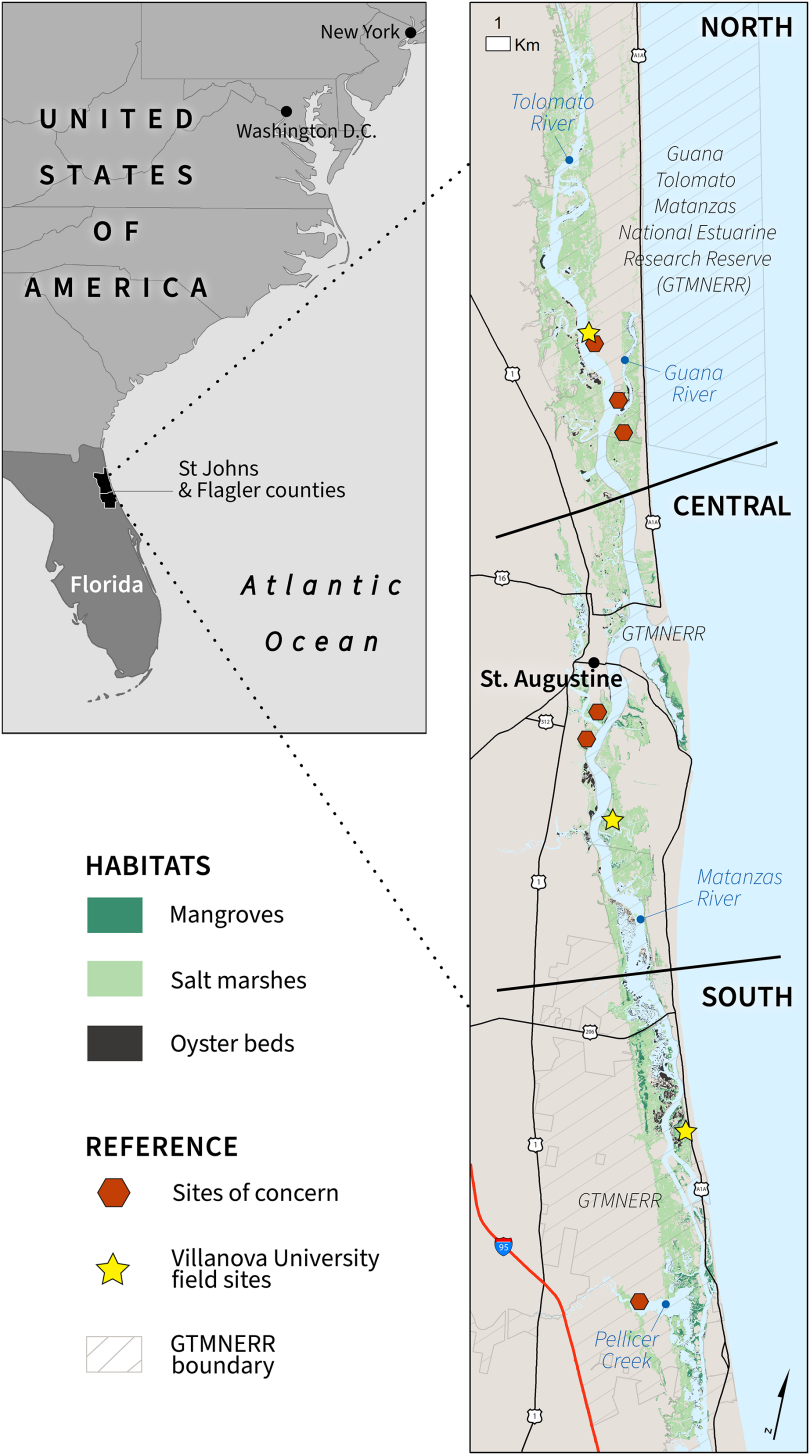
Study area map delineating the three estuarine habitats, major rivers, and GTMNERR boundaries.

We carried out research in the Intracoastal Waterway (ICW) near Saint Augustine, Florida, which is part of the Guana Tolomato Matanzas National Estuarine Research Reserve (GTMNERR) along Florida’s First Coast in St Johns and Flagler counties. GTMNERR, one of 30 NERRs in the United States, is in the Florida Upper East Coast Drainage Basin, and includes approximately 76,000 acres of publicly owned forested uplands, tidal wetlands, estuarine lagoons, and offshore seas. The greater St. Augustine area is home to about 16,000 people, with approximately 300,000 visitors annually to the GTMNERR’s visitor center, beaches, and trails. To segment the habitat classification and compare the analytical results, we split the GTMNERR into three sections (north, central, and south) of equal size. These areas were compared in the spatial analysis to highlight trends among the variable ranks and summary metrics (Fig. 1).

The GTMNERR is composed of the Matanzas River estuary in the south and the Tolomato and Guana River estuaries to the north. The vessel traffic of the ICW utilize both the Tolomato and Matanzas estuaries, and this riverine system merges near St. Augustine before emptying into the Atlantic Ocean. The Guana River runs parallel to the Tolomato River and was not considered in this study because of the lack of significant wave action from winds and boat wakes. Since the early 20th century, the physical environment (*e.g*., hydrology, increasing mangroves, *etc*.) of the Guana Tolomato Matanzas estuary has been modified by water management systems, including dikes, wells, dams, and ditches for drainage.

The US Coastal Barrier Resource System (CBRS) presents habitat management opportunities. Federal expenditures to develop in these areas are restricted to conserve the environment and wildlife of these ecologically critical coastal barriers. There are four CBRS units in the GTMNERR vicinity: Guana River, Usina Beach, Conch Island, and Matanzas River. Florida’s First Coast region is also part of the Atlantic Flyway, which provides habitat for several avian species not found elsewhere in the United States, including the Snail Kite, Limpkin, and Roseate Spoonbill. There are also two important bird areas (IBAs) designated at the Florida state level (Donald et al., 2019), which highlight avian biodiversity inside the Reserve: The Guana River (north) and Matanzas River and Inlet (central and south) (https://www.audubon.org/important-bird-areas/state/florida).

### Stakeholder engagement, research questions, and terminology

National Estuarine Research Reserves provide long-term research, stewardship, and education opportunities. The GTMNERR’s mission is to conserve natural biodiversity and cultural resources by using research and monitoring results to guide science-based stewardship and education. Resource managers, including those at the GTMNERR, depend on spatially explicit information to prioritize management activities throughout this dynamic ICW and estuary. Near-term research and management priorities for the GTMNERR (2022) include: “…habitat mapping, sediment transport, hydrodynamic, and ecosystem service studies to investigate the benefits and tradeoffs of different management options.” Land managers seek proactive techniques to promote coastal wetland resilience as coastal ecosystems continue to suffer the onslaught of rising seas and big storms. Our research responds to this fundamental need by asking the question: What are the feasible options to analyze existing data and identify suitable areas for restoration projects?

In partnership with the GTMNERR, Villanova University organized two workshops to engage regional subject matter experts and stakeholders as part of a NERRS Science Collaborative Catalyst grant. Subject matter experts and stakeholders represented St. John’s Water Management District, Anastasia Mosquito Control District, Florida Department of Environmental Protection Aquatic Preserves, Flagler County, U.S. Army Corps of Engineers, the Florida Fish and Wildlife Commission, North Florida Land Trust, and several universities. Initial workshops were held virtually (due to COVID-19) in February and March 2021 to highlight regional habitat restoration case studies and orient participants to the coastal vulnerability assessment. During this workshop, we explored various management timeframes and applications while getting a sense of the “restoration culture” in northeast Florida. A second, in-person workshop was organized in September 2021 to discuss management feasibility considerations and highlight sites of concern. Visits to potential restoration sites served to ground truth some of the variables in the coastal exposure index. The in-person workshop also facilitated further discussion of restoration strategies and identifying knowledge and resource gaps associated with each. Early on, stakeholders called on the research team to characterize boat wake energy in the ICW. It is a known driver of erosion throughout GTMNERR, as supported by recent literature to collect data and apply numerical models on boat wakes (*e.g*., Dahl, 2016; Safak et al., 2020a; Forlini et al., 2021). While recent studies have advanced our understanding of the complex interplay between vessel traffic and coastal ecosystems, such as quantifying the impacts of boat wakes at different scales based on field experiments (Forlini et al., 2021; Safak, Angelini & Sheremet, 2021), there was scant information about the relative intensity of boat wake exposure in the narrow creeks and channels of GTMNERR, a data gap we hoped to fill with this assessment.

Flooding and erosion caused by boat wakes and other disturbances (*e.g*., shoreline hardening, mangrove encroachment, *etc*.) pose substantial risks to vulnerable estuarine habitats in GTMNERR. We position this research to enhance ecological “resilience” in the region, *sensu* Holling (1973): the ability of a system to maintain its structure and functionality despite a shock. While ecotonal shifts and human impacts will likely continue, and at a greater pace and intensity, our objective is to screen a large area for opportunities to restore critical habitats and maintain the suite of vital benefits flowing from nature and people. Coastal resilience aims to bolster “the ability of systems to absorb changes of state variables, driving variables, and parameters, and still persist” (Holling 1973, p. 17). To the end, we ask: can the restoration of salt marshes, oyster reefs, and mangroves help to maintain their normal patterns of nutrient cycling, filtration, elevation maintenance, and other ecosystem services after being subjected to disturbance?

As confirmed during the engagement workshops, *exposure*, *hazard*, *vulnerability*, and *risk* are often conflated issues. For clarity, we define these terms as they apply to this coastal-estuarine assessment. A *hazard* caused by storm events impacting the coastal zone can result in inundation and erosion. Erosion and flooding may negatively affect people and wetland communities, so we refer to them as “hazards.” We refer to “exposure” as the coastal area where hazards may occur. Physically vulnerable populations (human and wetland communities) are highly exposed to coastal hazards. The *risk* is the potential consequences of erosion and flooding (*e.g*., mortality, economic and ecological damages). Here, we describe a coastal exposure index to quantify and map the relative risk of hazards acting on the physical environment of the GTMNERR.

### Coastal exposure index

To identify the areas most exposed to coastal hazards relative to the GTMNERR vicinity (Fig. 1), we used the InVEST coastal vulnerability model (naturalcapitalproject.stanford.edu). The model generates a coastal exposure index based on a user-defined set of variables to map the relative risk of shoreline to coastal hazards (Sharp et al., 2018). In addition, the InVEST approach builds on similar indices that account for the geomorphic characteristics of the study region (*e.g*., Gornitz, 1990; Palmer et al., 2011; Liquete et al., 2013) by explicitly considering the role of biogenic habitats in coastal protection and identifying where these habitats have the greatest potential to reduce risk. Coastal vulnerability model outputs can be compared across different conservation and development scenarios by accounting for anticipated changes in the distribution of infrastructure (*i.e*., built, natural) when framing risk (Arkema et al., 2013; Silver et al., 2019).

We subdivided the GTMNERR area into morphologic segments at 50m spatial resolution intervals. To convert these segments into a spatially explicit (mapped) coastal exposure index, we assembled data variables that serve as proxies for complex shoreline processes influencing coastal erosion and flood risk. The resulting exposure index (EI, Eq. (1)) expresses the coastline’s relative erodibility and degree of resistance to hazards by drawing on semi-quantifiable variables comprising six structural components. Based on the local factors identified by experts for GTMNERR, the EI variables selected include (1) coastal relief, (2) geomorphology, (3) the distribution of estuarine habitats, (4) wind exposure, (5) boat wake exposure, and (6) storm surge potential. For each coastline segment, six variables were weighted equally and aggregated using a geometric mean:

(1) $$EI = {\left( {{R_{Relief}}{R_{Geomorphology}}{R_{Habitats}}{R_{Wind}}{R_{BoatWakes}}{R_{SurgePotential}}} \right)^{{1 \over 6}}}$$

where *R* is the rank (1–5) determined for each of the biophysical variables indicated as subscripts.

We acquired spatial and tabular data from field surveys, expert interviews, participatory mapping, and satellite image classification to calculate these six variable ranks. Using observed and modeled data, we assembled information for the entire study area using a combination of absolute and relative values (Table 2). For the latter, numerical variables were assigned an exposure ranking based on data value ranges. We grouped all variables into three categories (low = 1.0–2.5, middle = 2.5–3.5, and high >3.5). Given that the geometric mean resolves to the same scale as the inputs, the rationale is that these three categories highlight which ranks drive exposure (*i.e*., the highest category). Ranks 1–2 contribute to lower exposure and 4–5 to higher overall EI scores. The following sections describe how these data were sourced, prepared, and used within the modified InVEST coastal vulnerability modeling framework. Detailed metadata describing the spatial inputs can be found in Table S1. This includes source, scale, resolution (spatial and temporal), date of acquisition, and how these data were used in the model.

**Table 2:** Six biophysical variable ranks of the coastal exposure index (EI). Ranks for relief, wind exposure, and surge potential are based on the distribution of values for these variables across all coastal segments in the GTMNERR study area.

Variable rank	Very low exposure rank (1)	Low exposure rank (2)	Moderate exposure rank (3)	High exposure rank (4)	Very high exposure rank (5)
Relief (average meters above MHHW)	>1.200 (>75^th^ percentile)	0.575–1.200 (50^th^ to 75^th^ percentile)	0.225–0.575 (25^th^ to 50^th^ percentile)	<0.225 m (<25^th^ percentile)	–
Geomorphology	Rocky; high cliffs; seawalls	Medium cliff; bulkheads and small seawalls	Low cliff; alluvial plain; revetments, rip-rap walls	Barrier beach; sand beach; mud flat; delta; estuary	–
Estuarine habitats	Mangroves	Marshes; oyster reefs	–	–	No habitat
Wind exposure (REI score)	<186.2 (<20^th^ percentile)	186.2–406.2 (20^th^ to 40^th^ percentile)	406.3–922.7 (40^th^ to 60^th^ percentile)	922.8–2,183.8 (60^th^ to 80^th^ percentile)	>2,183.8 (>80^th^ percentile)
Boat wake exposure (max height)	Shin (0.3 m)		Knee (0.6 m)	Waist (1.0 m)	
Surge potential (category three storm inundation height)	–	<13 ft (<33^rd^ percentile)	13–15 ft (33^rd^ to 66^th^ percentile)	>15 ft (>66^th^ percentile)	–

### Relief

Comparing differences in the elevation along the coast (relief) are critical to assess the risk of flooding and estimating its inward extent. We used pre-Hurricane Matthew LiDAR survey data processed and resampled by the University of Florida at 50m^2^ spatial resolution. We then applied a 2 km focal mean to obtain neighborhood elevation levels for each coastal segment. This neighborhood search distance was selected following sensitivity testing (radius = 1–5 km at 500 m increments) to ensure that the model did not capture distant elevation values from the coastline, which is less likely to influence risk during a flood event. Average elevation values were ranked 1 to 4 based on the distribution and using quantiles (75/50/25^th^ percentiles). We then compared the relative ranks for relief to available elevation measurements from Villanova University’s project field sites and in other areas of GTMNERR. There was good agreement (*R* = 0.93) between the empirical elevation data and the focal statistic (neighborhood mean) from LiDAR data (Fig. S1).

### Geomorphology

Rugged and hardened shorelines are less prone to erosion and inundation than beaches, mud flats, and estuaries. We adopted an approach for ranking coastal exposure based on a geomorphology variable introduced by Hammar-Klose & Thieler (2001). The NOAA Environmental Sensitivity Index (ESI; https://response.restoration.noaa.gov) provides a national summary of coastal resources that are at risk if an oil spill occurs and the relative sensitivity of shorelines to such an event. Most common in the GTMNERR study area is code “10A”, which according to the ESI rankings list, is classified as estuarine: salt- and brackish-water marshes. Further, NOAA describes the 10A shoreline as “sediments composed of organic muds except on the margins of islands where sand is abundant” and “common behind barrier islands and along the outer coast.” We initially assigned the highest rank score (5) for areas where this geomorphology type exists, indicating the greatest exposure category.

Notably, the ESI dataset does not provide a geomorphic classification of the coastal region protected by structures (*i.e*., seawalls, revetment, riprap, or other ‘grey’ solutions). Thus, for simplification, we assumed that where structures were present, they replaced the natural geomorphology described by the ESI. We incorporated the St. Johns County Shoreline Classification product (S. F. Eastman, 2014–2017, personal observations) to account for the protection provided by complex structures and other shoreline armoring. To create a wall-to-wall geomorphology ranking, we first used linear units of the ESI to define the coastline. We then extracted geomorphology scores 1–5 based on the US national geomorphology classifications from Arkema et al. (2013). The model uses a weighted average to score the rank for segments with multiple geomorphology types in the vicinity of one 50-meter segment. In some instances, this variable had floating point values (*e.g*., 2.5). Based on local knowledge and fewer shoreline types in the region, we adjusted the max rank from 5 to 4 to shrink the variable scoring range so that beaches, for example, compared to seawalls, do not overweigh the importance of geomorphology in the index.

### Estuarine habitats

Natural habitats play an important role in decreasing the adverse effects of coastal hazards such as shoreline erosion and damage to coastal communities (Manis et al., 2015; Guannel et al., 2016; Arkema et al., 2017). Estuarine habitats (marshes, mangroves, and oyster beds) can dramatically reduce wave heights in shallow waters, stabilize shorelines to encourage the accretion of nearshore sediments, and serve as natural breakwaters to dissipate wave energy (Scyphers et al., 2011; Shepard, Crain & Beck, 2011; Barbier, 2016; Doughty et al., 2017). A unique feature of the InVEST coastal vulnerability model is the ability to input information about the distribution of natural habitats that provide coastal protection services. Beginning with oysters, the “Oyster Beds in Florida” is made available from FWC-FWRI (Florida Fish and Wildlife Conservation Commission-Fish and Wildlife Research Institute) and represents the best available inventory of oyster data in Florida. We filtered out polygons where the oyster condition was either noted as “dead” or “mostly dead” and smaller patches based on a minimum area of 50 m^2^. The large oyster rakes in the north Reserve were considered dead oyster reefs and removed from the consideration in the model. These patch size and habitat quality conditions are unlikely to provide significant coastal protection (*sensu* Arkema et al., 2013).

Conversations with local partners familiar with the ecology of GTMNERR revealed that new mangrove colonies (*i.e*., northward movement likely due to climate change) are not reflected in existing state-level habitat maps of mangroves provided by FWC-FWRI (https://geodata.myfwc.com/search). We improved the existing Florida habitat inventory for two estuarine habitats (mangrove and salt marshes) using a supervised image classification technique. A statistical model, Random Forest, was applied to detect previously unmapped mangrove colonies inside GTMNERR. Random Forest is a popular machine-learning algorithm that uses decision trees to classify high-resolution imagery based on training data (see Data S1 for complete methods). A confusion matrix used to estimate type I and II errors revealed an overall model accuracy score of 91%. Larger patches of mangroves (>100 m^2^) were systematically reviewed for misclassifications (occasionally classified as upland forest by the model) and then “ground-truthed” by the team using recent aerial photos on Google Earth. A similar spatial filtering routine was applied to mangrove habitats (<50 m^2^) because juvenile and fragmented mangroves are less likely to provide substantial coastal protection services.

The magnitude and extent of natural protection provided by habitats in the coastal zone is a function of their age, density, quality, and other biological characteristics (Ruckelshaus et al., 2016). To combine the three estuarine habitat layers for generating one variable rank in terms of their combined protection, the model requires two parameters for each habitat type: (1) protection distance and (2) rank score. The former specifies a threshold distance for coastal protection afforded by the habitat, and the latter parameter indicates the protective capacity of the habitat relative to others. Next, the model computes the natural habitat exposure ranking (Eq. (2)) based on various possible combinations of natural habitats. The rank for the habitat variable of each shoreline segment was calculated as follows:

(2) $${R_{Hab}} = 4.8 - 0.5\; \sqrt {(1.5\; max_{k = 1}^n{{\left( {5 - {R_k})} \right)}^2} + \; \sum _{k = 1}^n{{(5 - {R_k})}^2} - max_{k = 1}^n{{\left( {5 - {R_k})} \right)}^2}}$$

where the habitat with the lowest rank is weighted 1.5 times higher than all other habitats present near a segment. A ${R_{Hab}}$ score closer to “1” offers the greatest protection, “4” the least, and “5” designates no protection afforded by habitat (Sharp et al., 2018). This equation takes a precautionary approach to estimate the value of shoreline protection by maximizing an account of the protection services provided by all the habitats that front a shoreline segment or are within a reasonable distance.

### Wind exposure

Heavy winds can result in high surges and contribute to powerful waves if they act on an area for an extended period. Climatic forcing conditions modeled with the InVEST model include wind and wave exposure variables. However, we focused on locally generated waves from wind because the interior ICW is sheltered mainly from oceanic waves. The wind exposure variable is used to score coastal segments based on the relative exposure to strong winds. It requires historical weather records over a long time (20+ years) and reanalyzing these data to better capture trends in wind-generated waves throughout the estuary.

There was no publicly available wind information for the northern section of GTMNERR, and we noted inconsistencies in the readings from instruments located in the south. We ultimately acquired wind data from two sources: (1) NOAA Buoy (channel entrance, central section) and (2) modeled wind data from OpenWeatherMap.org for three locations in the northern and southern sections (St. Augustine airport, Murat Point, and Marineland/Matanzas Shores). These sources provided climatic data for the sheltered areas inside the estuary based on more than 30 years of wind records (1979–2019), including wind speed (m/s) and direction (deg) at 60-m intervals. The resulting wind fields were processed in Python to generate wind roses and scatter plots. We analyzed these plots (Fig. S2) to identify abnormal peaks in the wind speed measurements during major storm events. For example, Hurricane Gabriel in 2001 generated winds of greater than 30 m/s. Ultimately, these outliers were removed from the wind statistics and formatted similarly to the InVEST input table described by Sharp et al. (2018).

To calculate the wind exposure variable rank, we used the Relative Exposure Index (REI) scoring approach defined by Keddy (1982). First, the model uses spatial calculations to estimate fetch (how far wind blows over an area) for a given shoreline point by casting rays outward in 16 directions and measures the maximum length of a ray before it intersects with a landmass (*F*_*n*_ in Eq. (3)). REI is computed by taking the highest 10% wind speeds from the nearest record of measured wind speeds, dividing the 360 degrees compass into 16 equiangular sectors, and combining the wind and fetch characteristics in these sectors as:

(3) $$REI = \sum\limits_{n = 1}^{16} {{U_n}} \;{P_n}\;{F_n}\;$$

where: *U*_*n*_ is the average wind speed, in meters per second, of the highest 10% wind speeds in the n^th^ equiangular sector; *P*_*n*_ is the percent of all wind speeds in the record of interest that blow in the direction of the n^th^ sector; and *F*_*n*_ is the fetch distance in meters, in the n^th^ sector. Finally, we assigned a rank value (1–5) according to the REI scores and based on the distribution’s 20/40/60/80th percentiles.

### Boat wake energy

Boat wakes cause substantial coastal erosion in the ICW of the GTMNERR (Price, 2005; Safak et al., 2020a). Wave energies from boat wakes are event-dependent and influenced by the vessel length, water depth, channel shape, and boat speed, among other variables (Glamore, 2008). Wakes are most impactful in shallow and narrow waterways because wake energy does not have the opportunity to dissipate over long distances (Fitzgerald, Hughes & Rosen, 2011). To quantify boat wake exposure, we drew on a network of local experts that consisted of the GTMNERR, conservation NGO staff, and sportfishing and water tour operators. Online interviews were conducted using a participatory mapping approach (PGIS) created in the Survey123 application to identify hotspots and critical gaps in boat wake exposure throughout the ICW. The consultations resulted in *n* = 128 geolocated annotations to provide a qualitative indication of wake climate (shin, knee, or waist height; 0.3, 0.6, 1.0 m, respectively). Next, we created Voronoi polygons, a spatial analysis technique used to allocate point data to the most proximate linear segment, which in this case were the relative heights specified during expert elicitation (Fig. S3). The spatial coverage of the PGIS for tidal creeks and tributaries was sufficient, but there were gaps in the main channel of the ICW, especially along the Guana and Matanzas rivers (central and south sections of GTMNERR).

The Automatic Identification System (AIS) is used by the US Coast Guard and others to track boat location, speed, length, sailing line, and hull type (Tetreault, 2005). Drawing on two years of AIS data collected at 1-m intervals (https://marinecadastre.gov/ais), we summarized boat density based on the proportion of vessels traveling through a grid of 1 km hexagon cells in three speed categories: slow (<10), plowing (10–20), planning (>20 kt). Fonseca & Malhotra (2012) suggest that plowing speeds (~10 knots) produce the greatest wakes heights and potential for erosion based on observations in the Atlantic Intracoastal Waterway of North Carolina. To examine how the accumulation of boat-induced waves varies based on vessel density and speed, we analyzed vessel traffic as a function of traveling speed and location. First, we split the area of interest into two zones: (1) the main channels of the ICW and (2) side channels and tidal creeks. Published studies (Sorensen, 1973; Bilkovic et al., 2019) indicate that recreational vessels within 150 m (~500 ft) of the shoreline can produce waves large enough to result in significant shoreline erosion. We used focal statistics in GIS–with a fixed search radius (moving average)–to generate a distance decay and relative ranks (33/66^th^ percentiles) for all hexagonal cells inside a 1 km neighborhood. We applied boat wake exposure ranks between 1 to 3 for sheltered coastline (*i.e*., blocked by a landmass or narrowing of the channel) and 3 to 5 for exposed areas. When scoring boat wakes in the main channels, we defaulted to the AIS estimates if there was a mismatch in the information collected during the expert judgment (PGIS) phase. Overall, relative boat wake exposure was ranked 1 to 5, corresponding to summary scores of vessel density, speed, and distance to shore in zone 1 and shin-to-waist height waves as recorded in the PGIS for zone 2.

### Storm surge potential

Models of storm surge flooding vulnerability provide critical information to residents of hurricane-prone coastal areas such as Florida’s First Coast and assist in evaluating the risk of the storm surge hazard. Surge elevation is related to wind speed and direction, but also the length of time wind acts on relatively shallow areas (Sharp et al., 2018), such as the main channel of the ICW. In some situations, the risk of storm surge can extend long distances inland from the immediate coastline. In the absence of models to project surge elevation during different storm events, coastal vulnerability assessments (*e.g*., Arkema et al., 2013; Silver et al., 2019) use distance to the continental shelf as a proxy for storm surge potential. Given that this is an ICW analysis and surge model results exist for the entire study area, we selected a Category 3 storm scenario using the Sea, Lake, and Overland Surges from Hurricane (SLOSH) model developed by NOAA and the National Weather Service (https://nhc.noaa.gov/surge/slosh.php). For most of the US, the SLOSH model provides estimates of inundation height in feet above the ground, and, for all coastal segments within the GTMNERR study area, the mean value was 13.9 feet (range = 2–18 ft). SLOSH inundation height values were then allocated to the nearest shoreline pixel. Given the narrow shape of the overall distribution, we ranked the surge potential variable between 2 and 4 using the 33^rd^/66^th^ percentiles of the distribution.

### Habitat role in natural protection

In the context of multiple valuation methods to assess the protective services of wetland habitats (Barbier, 2016), the relative role of habitats can be a helpful metric for designing coastal plans and developing specific recommendations for rehabilitation and restoration strategies (*e.g*., Ruckelshaus et al., 2016; Silver et al., 2019). To quantify and map the role of habitats in natural protection, we calculated the difference in exposure index scores with and without the presence of estuarine habitats. As an illustration, the habitat role scores would be identical for two sections of shoreline backed by similar salt marsh habitats only if all other EI variable ranks were equal. This approach serves to tease out the protective potential of habitats in the context of other coastal exposure factors and offers a roadmap for management. We assessed the individual and combined role of mangroves, salt marshes, and oyster beds to identify where these habitats contribute most to shoreline protection.

### Spatial analysis

The GTMNERR study area, in particular the main channel of the ICW, is long and narrow. It became a challenge to share detailed results using static maps throughout the engagement and communication process. To address this, we produced an interactive results viewer (http://cons.scienceontheweb.net/ewe), which was first deployed during the February and March 2021 workshops. Drivers of exposure (*i.e*., variable ranks 4 and 5) can be displayed by selecting an area of interest and analyzing the bar charts. In addition to providing more substance to the analytical results, the viewer offers reference information such as boating features (ramps, marinas, launches), CBRS units, IBAs, and sites of concern. We also conducted a spatial analysis to identify the most critical areas for habitat protection and enhancement in the context of six sites of concern identified by GTMNERR stakeholders (Fig. 1; Table 3). The three criteria for the highest protection/enhancement need were shoreline segments that (1) transition to the highest exposure level if habitats were lost, (2) stakeholders flagged as a site of concern, and (3) are not part of the CBRS or GTMNERR network of protection. The rationale for the third criterion is that these areas are more likely to be developed and, therefore, more difficult to propose and implement habitat restoration strategies such as living shorelines or mangrove rehabilitation.

**Table 3:** Synthesis of findings for sites of concern based on the coastal exposure index and habitat role metrics produced by the InVEST coastal vulnerability model.

Drivers of exposure and high habitat role in natural protection by GTMNERR section		Networks				Potential strategies for elevation maintenance
Sites of concern		Transition to highest exposure category (%)	CBRS	GTM NERR	IBA	
**NORTH** Geomorphology, relief; Few mangroves, marshes, and oysters in tidal creeks						
**Big Mama**		0	✓•	✓•	✓•	Mangrove establishment (minimal natural mangrove recruitment but relatively intact marsh habitat)
**Guana Peninsula**		0	✓•	✓•	✓•	Living shorelines (because of oysters in creeks and less boat traffic)
**Hat Island**		13	✓	✓	**✗**	Living shorelines, thin-layer placement
**CENTRAL** Boat wakes, surge potential, relief; Mangroves and marshes in the back channels						
**N-312**		27	**✗**	✓	**✗**	Mangrove establishment
**S-312**		73	**✗**	**✗**	**✗**	Living shorelines, landform modification
**SOUTH** Relief, surge potential, wind exposure; Mangroves, marshes, and oysters in the widest parts of the main channel						
**Pellicer Creek**		0	✓•	✓•	✓•	–

**Note:**

Conservation and NERR networks are presented in the context of sites of concern where **✗**, no overlap; ✓, partial overlap; ✓•, full overlap (land and water).

## Results

### Relative exposure to hazards

**Figure 2:**
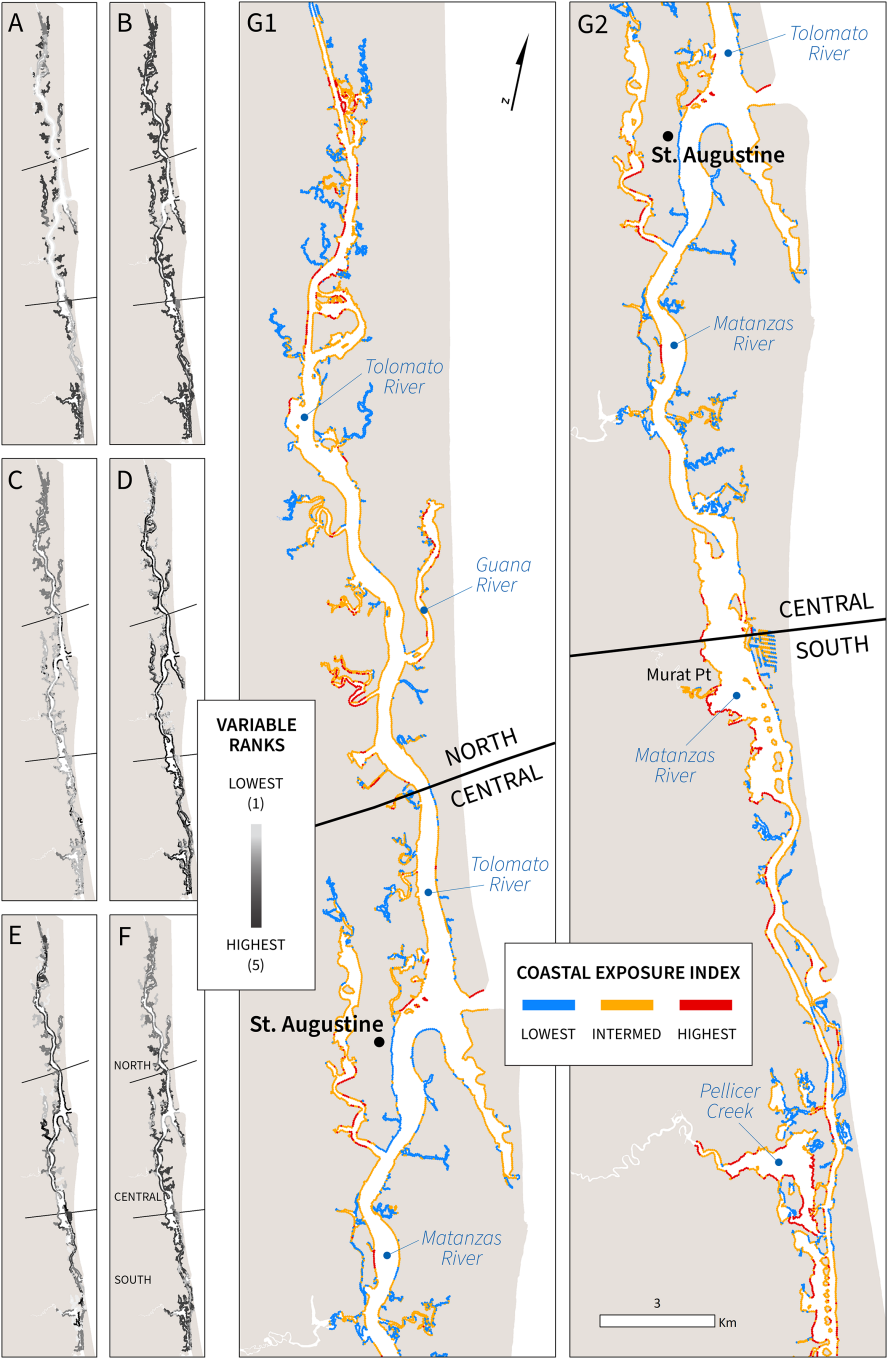
Spatial distribution of individual variable ranks and exposure index scores for GTMNERR: (A) relief, (B) geomorphology, (C) estuarine habitats, (D) wind exposure, (E) boat wake exposure, (F) surge potential, (G1–2) exposure index.

**Figure 3:**
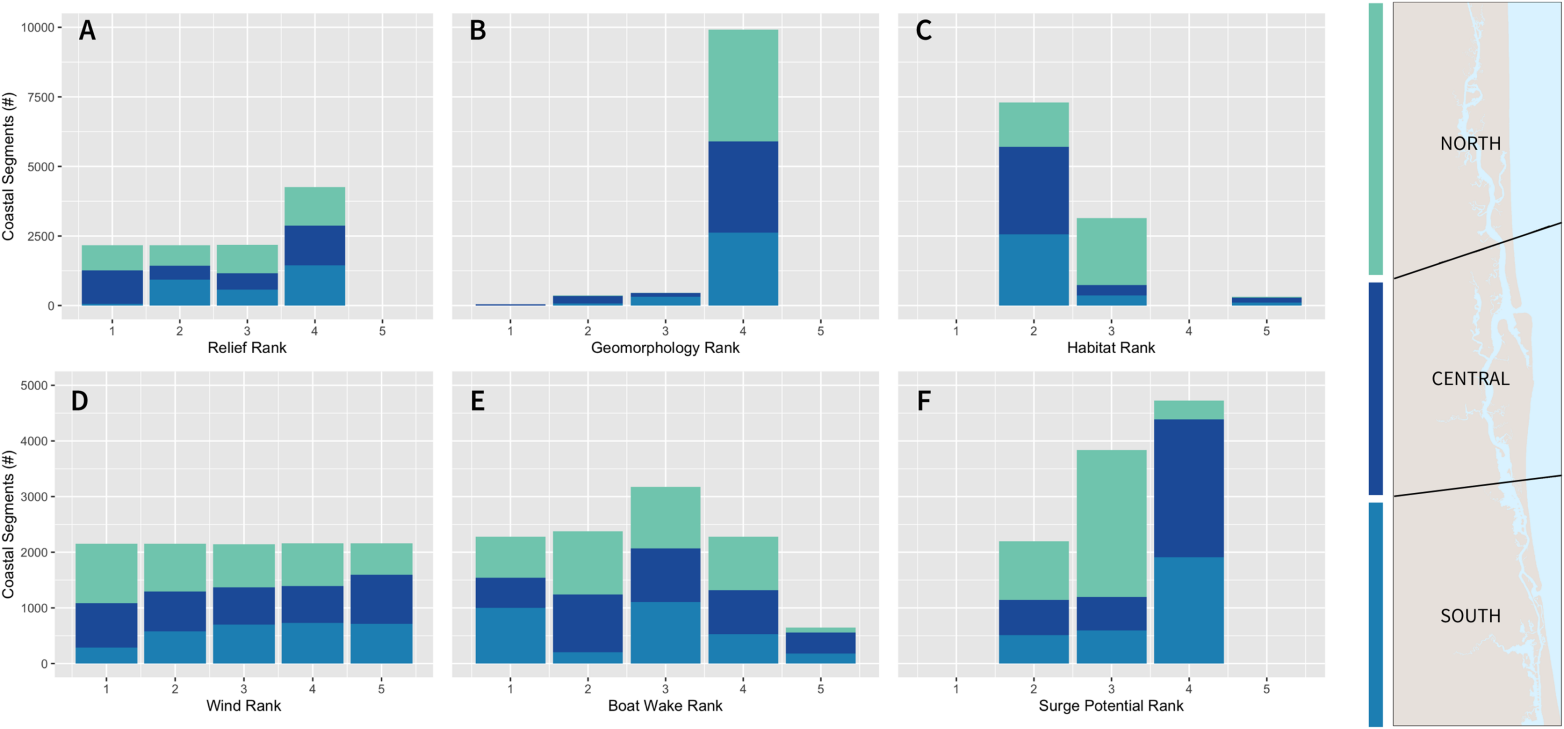
Bar charts of exposure index variable ranks by subregion (north, central, south; *n* = 4,031, 3,712, 3,018, respectively). Floating point scores for geomorphology variable were rounded up to the nearest integer bin for the purpose of this visual.

A total of 538 km of estuarine shoreline were screened, evaluated, and classified as three categories of coastal exposure (lowest, intermediate, and highest; Fig. 2G). All six variables in the model passed tests for collinearity with Pearson’s correlation scores *r* ≤ 0.42 (Fig. S4). Exposure index (EI) scores above 3.295 were assigned to the highest category (>75^th^ percentile of the distribution of values), and these greatest exposure areas were found throughout the Reserve. High EI scores were most common in areas fully exposed to wave action (wind-generated and boat wakes) and not currently backed by estuarine habitats. This greatest exposure category was also found in the main channel of the ICW, corresponding to coasts with less stable coastal geomorphology (muddy/sandy shoreline, rank = 4) and relatively low neighborhood elevation (<0.225 m; Figs. 2A and 2B). Geographically, the greatest proportion of coastline with the highest exposure index scores was in the northern and southern sections of the Reserve (9% and 14%, respectively) compared to only 4% in the central. In the north, the model determined these exposure hotspots to be primarily driven by less stable shoreline geomorphology and limited protection afforded by the current distribution of estuarine habitats (Figs. 3B and 3C). A combination of lower elevation areas, stronger surge potential, and substantial sections of the coastline oriented towards locally generated waves (prevailing winds) were the most critical factors contributing to high exposure levels in the southern section of GTMNERR (Figs. 3A, 3D, and 3F).

Waves with longer periods are more powerful, causing more severe inundation and higher rates of coastal erosion over time (Holthuijsen, 2010). Boat wakes have longer periods than wind-generated waves, and thus cause more damage (Fitzgerald, Hughes & Rosen, 2011; Bilkovic et al., 2019). The entire study area is sheltered from the open sea and not subject to oceanic waves. Inside the main channel of the ICW, exposure scores were more varied (Fig. 2). The model accounts for channel width, shoreline orientation and predominant wind direction, and the presence of small islands serving as natural breakwaters. These variables, along with powerful boat-generated waves, can add to local forcing conditions and influence this variation in exposure. For example, Murat Point is more exposed relative to similar sections of the coast on the opposite (east) side of the channel (Fig. 2G). The dominant wind direction based on historical data suggests a north/northeast origin (0–23 degrees; Fig. S2). For shoreline segments exposed to these directions and subject to large fetch length (*e.g*., wider portions of the main channel), there are likely to be consistently higher wave heights from wind-generated waves (wave exposure rank = 4 or 5). Boat wake exposure, driven by a combination of high-density vessel traffic and consistent plowing speeds between 10 and 20 knots/s according to AIS vessel data, were also a factor for the highest exposure scores. The central section of the ICW, which consists of the largest area of the main channel and vessel traffic entering from the Atlantic, is subject to the greatest proportion of rank = 5 (highest boat wake exposure), with observed wakes as much as waist high (1 m). The narrower sections of the central ICW, with substantial vessel traffic and not currently fortified by structures nor backed by biogenic habitats, were often categorized in the highest exposure category by the model. These coastal exposure hot spots near St. Augustine city (Figs. 2E–2G) are subject to wave overtopping, run-up, and long-term erosion where consistent wakes are generated by vessels traveling at medium speeds that enter the Matanzas River *via* the Saint Augustine inlet.

The potential for storm surge is greatest in the central and southern Reserve (Fig. 2F). As indicated by the NOAA SLOSH Maximum of the Maximum model, surge funnels through the narrower sections of the Matanzas River (central to south Reserve) and is predicted to manifest as inundation heights greater than 15 ft for the majority of coastline in these two sections. Specifically, the analysis of the storm surge variable shows that 67% and 63% of all coastal areas in the central and south sections, respectively, were scored as the highest surge rank (>66^th^ percentile; Fig. 3F). In contrast, only 9% of the north section had the highest surge rank score. Storm surge impacts (*i.e*., inundation) can be more extreme in low-elevation areas. The northern and southernmost sections of ICW were found to be some of the lowest-lying areas with a mean neighborhood elevation <0.23 m above the MHHW level (Fig. 2A). If high wave heights from storm surge reach the coastline already subject to other hydrodynamic processes such as wind-generated waves, it can magnify the impacts from flood overtopping and erosion. The greatest potential for storm surge (rank = 4) in combination with high wind exposure (class = 4 or 5) and lowest neighborhood elevation (relief level = 4) accounted for the majority of shoreline classified in the highest exposure level of the south section (50%) compared to only 6% and 3%, respectively, in the north and central. These areas of the southern GTMNERR were further analyzed in the context of land tenure and the consequence of future habitat loss (see section: ‘Spatial analysis for sites of concern’).

### Habitat role in natural protection

**Figure 4:**
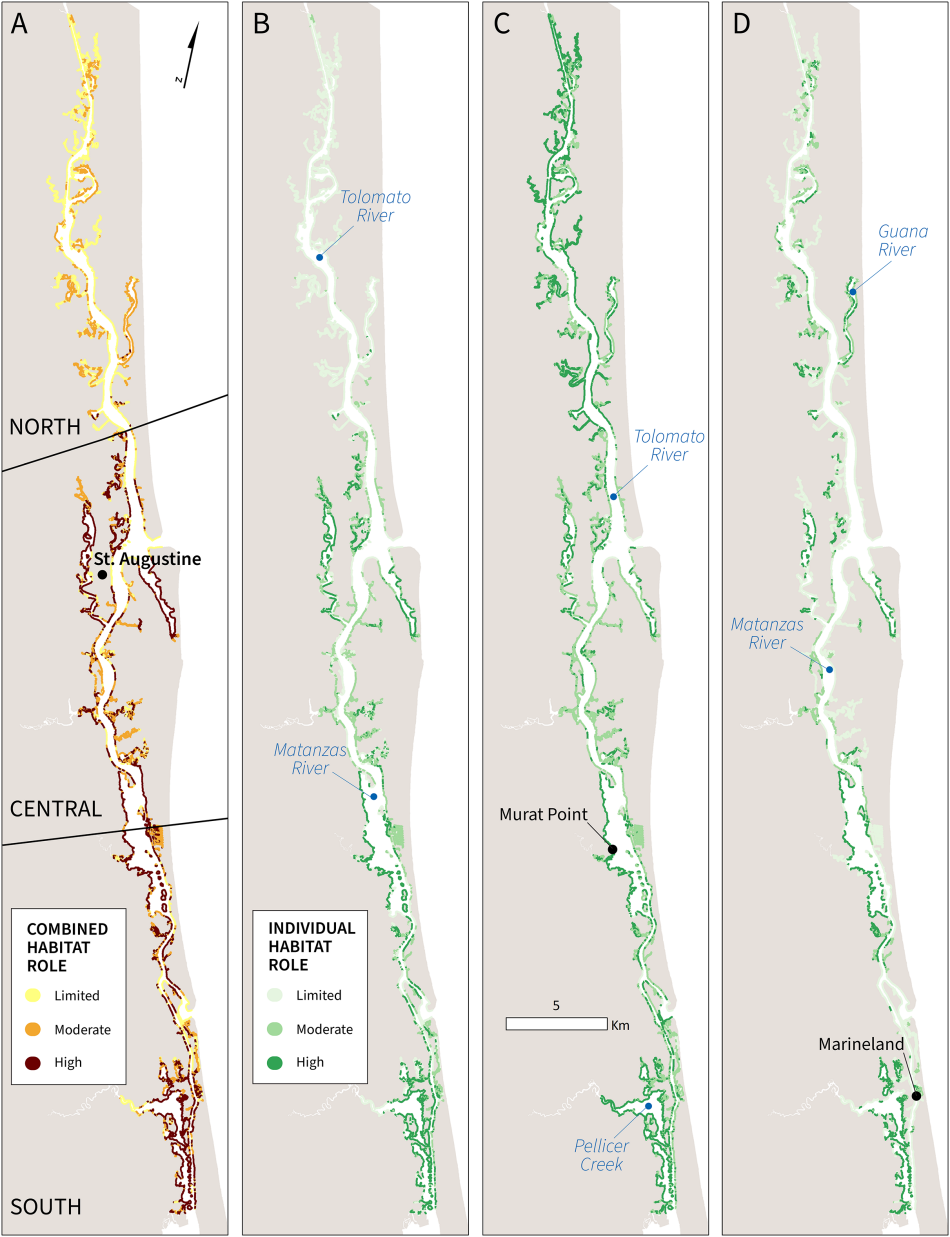
Maps of combined and individual habitat role scores for GTMNERR: (A) all habitats, (B) mangroves, (C) salt marshes, (D) oyster beds to highlight coastal risk-reduction benefits based on their distribution and relative to other factors that contribute to the exposure index.

The habitat role metric, derived from the coastal exposure index, indicates the distribution of natural protection these habitats provide. Findings suggest that the combined habitat role is highest in the central and southern sections of the Reserve (Fig. 4A), especially in the widest sections of the main channel where estuarine habitats reduce coastal exposure to boat wakes (Fig. 2E). In the highest exposure areas of the main channel of the ICW, three broad locations were identified by the model as providing high coastal protection service value: (1) part of the central GTMNERR, near St. Augustine city and southern Tolomato River, (2) the widest sections of the Matanzas River, and (3) Pellicer Creek near Marineland. These areas of the ICW and its back channels have high habitat role scores due to the presence of extensive biogenic habitats-mangroves and marshes-and high ranks (4 or 5) for other variables that contribute to exposure.

Light-colored areas of the maps (Figs. 4B–4D) indicate the limited individual role of habitats in reducing exposure considering other variables in the index. Overall, results suggest that current estuarine habitats offer the lowest levels of coastal protection in the north Reserve. The image classification techniques employed by this study to characterize the current distribution of estuarine habitats indicate that mangroves are less abundant in the northern section of the Reserve, perhaps due to slower infill or different marsh vegetation (Adgie & Chapman, 2021). Consequently, fewer biogenic habitats (especially mangroves and oyster beds) are distributed throughout this area to buffer wave action (Figs. 4B and 4D). Given that the current distribution of mangroves above 30 degrees latitude is sparse, the model suggests a more limited combined role for habitats in the north GTMNERR (Fig. 4A). Notably, the role of live oyster beds in natural protection is greatest in the narrow channels of the northern and southern sections, where wind and boat wake exposure are greatest (Fig. 4D).

### Spatial analysis for sites of concern

**Figure 5:**
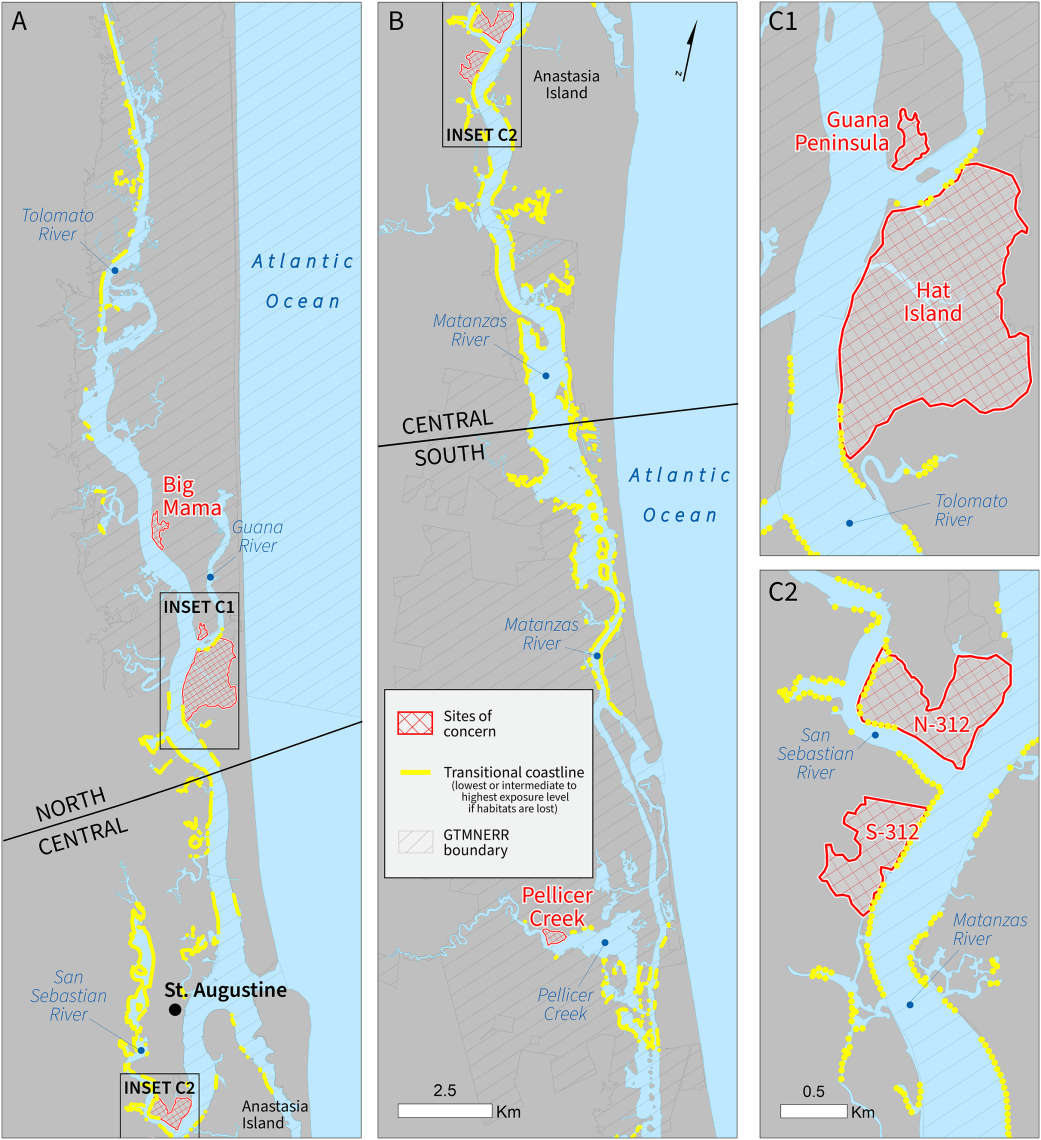
Marginal coastal protection benefits provided by estuarine habitats outside of CBRS units (yellow-colored coastline segments). These coastal areas are predicted to transition to the highest exposure level (index score > 3.295) if the current distribution of habitats were to be lost. Insets C1and C2 highlight four sites of concern (red double hatching) as identified during stakeholder workshops.

A total of 179 km of estuarine shores (33% of the study coastline) would transition to the highest exposure index category if habitats were lost. There is significantly less coastline dependent on habitats for reducing exposure in the north (26%) relative to the south and central (37–38%) (χ^2^ = 156.97, *p* < 0.01). These transition areas served as the starting point for our *post hoc* analysis to identify vulnerable areas that could benefit from elevation maintenance strategies (Table 4). Three areas were identified as meeting the three criteria (*i.e*., transition to highest exposure without natural protection, a site of concern, and not within the CBRS, GTMNERR, or IBA network: (1) Hat Island, at the northern section of the Reserve where the Guana and Tolomato Rivers converge (Fig. 5C) and (2 and 3) N-312 and S-312, where the San Sebastian River empties into the Matanzas River, just north of the bridge connecting St. Augustine city to Anastasia Island (Fig. 5D). The first area is highly exposed to climatic forcing conditions in the main channel, which is relatively narrow and subject to powerful waves from boat wakes and winds. There are extensive salt- and brackish-water marshes and oyster beds but no mangroves in this portion of the channel. The other two locations are also subject to wind and boat-generated waves, along with being at a relatively lower elevation. Ongoing restoration planning suggests the abrupt turn in the Matanzas River–just south of the State Road 312 bridge along the main navigation channel–plays a role in marsh loss on the banks of the ICW and broader stresses on wetland ecosystems. Here, the GTMNERR boundary only covers the waterways and not the upland areas (Fig. 5D). There are no CBRS units along this stretch of the ICW (Table 3), which has implications for future management.

**Table 4:** Summary of the four management options identified by land managers and stakeholders as most effective for maintaining or increasing wetland surface elevation at the GTMNERR.

Maintenance strategy	Definition	Location within wetland	Basic site requirements	Knowledge Gaps
Thin-layer placement	Dredge sediment is sprayed over large area of wetland to increase surface elevation incrementally (Wilber, 1992)	Edge or interior	Proximity to large channels for machinery access (Stagg & Mendelssohn, 2010); best applied when plants are dormant (Payne et al., 2021) or absent (VanZomeren et al., 2018); marsh dominated wetlands (Stagg & Mendelssohn, 2010)	General long-term impacts and subsidence potential; hydrology impacts; effect on mangroves within the marsh; impacts to invertebrates/microbes/algae/birds/ fisheries (VanZomeren et al., 2018)
Mangrove establishment	Mangroves are intentionally planted to allow natural accretion of sediment *via* mangrove root growth over time (Lewis, 2005)	Interior (Fillyaw et al., 2021)	Intact marsh habitat to increase seedling survival (Adgie & Chapman, 2021; Yando et al., 2019); annual tidal inundation ~30% (Fillyaw et al., 2021; Lewis, 2005); minimal natural mangrove recruitment (Fillyaw et al., 2021); low energy wave setting (Fillyaw et al., 2021)	Impact of marsh species diversity (Adgie & Chapman, 2021; Yando et al., 2019); differences in mangrove species’ elevation benefits and temperature thresholds (Osland et al., 2020); public perception of mangrove planting
Living shorelines	Stabilization of coastal wetland edge using natural materials, often oysters & vegetation (Morris et al., 2019)	Edge (Morris et al., 2019)	Oyster habitat suitability (turbidity, salinity, oxygen) (La Peyre et al., 2015); appropriate substrates (Bersoza Hernández et al., 2018; La Peyre et al., 2015); relatively low energy wave setting (Morris et al., 2019)	Enabling/limiting conditions for different types of shorelines (La Peyre et al., 2015); boat wake impacts; durability in energetic settings (Safak et al., 2020b)
Landform modification/berm redistribution	Redistribution of dredge spoil or shell rakes to restore functioning hydrology in wetland habitat behind the landform (Mora & Burdick, 2013)	Edge or interior (Mora & Burdick, 2013)	Proximity to large channels for machinery access; understanding of local hydrology (Lewis, 2005; Mora & Burdick, 2013; Pérez-Ceballos et al., 2020; Radabaugh et al., 2021)	Recovery time for marshes (Mora & Burdick, 2013) *vs*. mangroves (Lewis, 2005; Pérez-Ceballos et al., 2020); impacts on migratory birds; permitting process

## Discussion

Coastal zones worldwide are experiencing a combination of rapid population growth and climate change impacts from rising sea levels and more intense, frequent storms (Long et al., 2011; Reiblich et al., 2019). Site-specific assessments are needed to evaluate how natural and built environment modifications affect coastal vulnerability (*e.g*., Palmer et al., 2011; Liquete et al., 2013; Arkema et al., 2013). Through dialogues with land managers and stakeholders, we identified multiple factors contributing to coastal exposure and the areas of the GTMNERR that are most vulnerable to habitat loss due to low elevation, shoreline composition, and waves generated by wind and boat wakes. Next, we assessed the potential of three estuarine habitats to provide coastal protection services through a systematic screening of over 500 km of sinuous coastline. Finally, we evaluated sites of concern, including areas outside the Reserve, federally protected lands, and avian biodiversity hotspots where habitats currently provide substantial coastal hazard risk reduction benefits. The novelties of our study include the addition of boat wakes as a factor in coastal exposure and an assessment of two co-occurring coastal wetland habitats. The analytical approach targets wetland restoration pilot projects with specific elevation maintenance strategies that aim to manage human development and environmental conservation objectives in the context of a rapidly shifting ecotone.

### Adapting the CVI to the estuarine context

The persistence of estuarine ecosystems depends on their ability to gain sediment and keep pace with rising sea levels. Coastal habitats can accrete sediment and increase elevation, allowing the shoreline to adapt and maintain its relative position as the sea level rises (Gedan et al., 2011; Spalding et al., 2014; Manis et al., 2015), relying on intact upland habitat for inland migration (Crosby et al., 2016; Borchert et al., 2018; Cahoon et al., 2019). In line with the global trend, NE Florida’s coastal environment is experiencing rapid environmental change, evidenced by the ecotonal habitat shifts in the GTMNERR. Overall, the areas of highest exposure in the GTMNERR can be characterized as low elevation, sandy beaches or mud flats, absent of biogenic habitats to attenuate waves, and highly susceptible to storm surges and locally generated waves (Figs. 2G1 and 2G2).

Throughout the engagement process, stakeholders cited boat wakes as a significant cause of coastal erosion and habitat degradation along the ICW. Bilkovic et al. (2017) found boat-wake-induced erosion in Chesapeake Bay to vary based on vessel speed, length, hull shape, distance to shore, and shoreline composition (*e.g*., habitats and geomorphology). We accounted for the combined effect of locally generated waves from prevailing winds and boating activities to estimate relative wave heights in the GTMNERR coastal area. To the extent of our knowledge, boat wake exposure as a ranked variable has not been used in other assessments of coastal risk reduction. This novelty represents a feature enhancement of the InVEST coastal vulnerability model and increases our confidence in findings for the smaller creeks and narrowest waterways where AIS data are unavailable.

Areas of the main channel, specifically where it narrows (<500 m width) and with the most concentrated vessel traffic, were ranked highest by the model in terms of boat wake exposure (Fig. 2E). This trend was most apparent in the central section of the GTMNERR, with more than half (58%) of coastal segments ranked as 5, the maximum boat wake exposure level. These high-exposure areas also sit at low elevations and are subject to strong climatic forcing conditions from locally generated waves. Based on historical wind data (Fig. S2), waves originating from the N-NE tend to reach the most exposed shoreline in the main channel with a long fetch and minimal energy dissipation. For wider sections of the ICW and where the channel is far from shore, such as those near the St. Augustine Inlet, wind-driven waves are likely the most substantial factor in hydrodynamic forcing (C. Angelini, January 13, 2020, personal communication).

In addition to being a nursery for flora and fauna and the wildlife viewing benefits, biogenic habitats attenuate boat wake energy and prevent rapid bank erosion in estuarine waterways. For example, established smooth marsh grass (*Spartina alterniflora*) and the eastern oyster (*Crassostrea virginica*) decreased 67% of the wave energy from recreational boat wakes relative to mud flats in wave tank experiments (Manis et al., 2015). Both species are endemic to Florida’s Atlantic coast. Mangroves and marshes can reduce up to 90% of wave height, but mangroves need less area than marshes to provide higher levels of this service (Doughty et al., 2017). Our study accounts for the varying coastal protection services of different estuarine habitats by producing new maps of their distribution using a supervised classification of satellite imagery. High-value habitat areas of the GTMNERR–where there is a disproportionally higher contribution of these protection benefits relative to the five other exposure index variables–were then mapped (Figs. 4 and 5) and prioritized (Table 3) to illustrate that location does matter in the context of natural protection (Ruckelshaus et al., 2016). Yet, in the absence of more specifics about the biophysical characteristics (age, root depth, sediment type, *etc*.), our assessment of the contribution of mangroves, marshes, and oyster beds to risk reduction in GTMNERR can be considered precautionary.

Smaller fringe mangrove and marsh habitats provide limited services and are mainly helpful for erosion control and small wave attenuation (Pinsky, Guannel & Arkema, 2013; Guannel et al., 2015). Mangroves do not grow well in areas where there is regular wave action because they cannot settle and plant roots (Fillyaw et al., 2021). Once established, they prefer calm conditions, and future research could monitor newly established mangrove colonies and investigate their potential for wave attenuation in combination with other habitats. Here, we assumed all habitats with a contiguous patch area larger than 100 m^2^ provide coastal protection benefits. This is because smaller patches were difficult to detect during the habitat mapping. Nevertheless, it is encouraging that our habitat map indicates fewer large mangrove stands in the northern section of the GTMNERR, where the model predicts a lower risk of storm surge relative to the entire study area (Fig. 3F).

### Limitations and assumptions

The InVEST coastal vulnerability model is constrained by the quality of spatial input variables that comprise the exposure index (Sharp et al., 2018). Interactions between the six variables are not accounted for in the model, nor are potential changes in shoreline position or configuration. Still, the input variables can be repackaged to predict other flexible outputs such as inundation, flooding, and other coastal risks. For example, the variables used in the exposure index could be repackaged as subindices that describe the risk of inundation (flooding) or erosion. The former would include relief, surge potential, and natural habitats, while the latter could incorporate geomorphology, wind, and boat wake exposure information. For three physical variables (relief, wind exposure, and surge potential), statistical quantiles were used to bin the data based on the distribution of values and provide relative scoring ranks. The geometric mean calculation used to compute the index resolves to the same range as the input ranks (1–5) and highlights relative sensitivities along the coast. By mapping GTMNERR sites of concern based on shoreline characteristics against proximity to frequently traveled navigation channels, it could be possible to rank areas in terms of their overall susceptibility to erosion. For instance, unvegetated coasts with low vertical relief would rank highest for erosion concerns.

The presence of seawalls and other rigid structures can increase exposure to the neighboring coastline by amplifying wave energy (Silver et al., 2019). This edge effect is most apparent in the central Reserve (lightest grey segments in Fig. 2B). While possible to account for this effect as a new variable in the index, we relied on an incomplete inventory of shoreline armoring for the geomorphology rank. Consequently, the exposure index scores in the central region may be slightly conservative. In addition, we did not include sea level change as a variable in the coastal vulnerability model because this longer-term effect does not vary significantly within the study area according to historic tide gauges (Arkema et al., 2013). Although the GTMNERR coastline is likely to experience sea level change over time, the net rise or fall in water levels is not expected to vary spatially across the ICW. For estuarine habitats, the model assumes these input data reflect the current distribution of biogenic habitats and their capacity in coastal protection. Future changes in vegetation characteristics could be reflected as additional habitat scenarios evaluated by the model. GIS data on coastal erosion and accretion rates from damage assessments can serve to validate the model. Coastal-nearshore slope and sediment type would be helpful to inputs in a process-based wave model for exploring elevation maintenance using a modified marsh equilibrium model (Morris et al., 2002).

Quantifying boat wake climate in sheltered areas is challenged by the complex interplay between site-level factors such as nearshore bathymetry, sediment grain size, wind speeds, and shoreline orientation (Glamore, 2008; Fonseca & Malhotra, 2012; Zaggia et al., 2017). Data on these and other variables contributing to shoreline erosion and flooding from boat wakes do not exist for much of the GTMNERR area. Here, we used a qualitative ranking approach to estimate relative exposure throughout the estuary. Expert knowledge collected during the boat survey interviews suggests that wake heights can be substantially higher in waterski zones and other confined areas (C. Reed, May 11, 2020, personal communication). We identified proximate coastal areas to significant wake events where AIS-monitored vessels consistently operated at plowing speeds (10–20 knots). This information was then used to fill coincident gaps in coverage of the boat wake interview surveys (largest Voronoi polygons in Fig. S4). In ranking the boat wake exposure variable, we did not consider shoreline orientation to avoid double counting this effect and minimize its correlation with the wind exposure variable (*r* = 0.42).

### Elevation maintenance strategies

Wetland surface elevation maintenance strategies such as landform modification, living shorelines, mangrove establishment, and thin-layer placement offer promise to meet priorities for enhancing recreation, education, shoreline protection, allowing wetland migration inland and other ecosystem-service co-benefits identified by GTMNERR staff and stakeholders. To render our exposure index map useful, we held a series of workshops with regional stakeholders to ensure development and application were made with their input. In the first workshop, we heard restoration stories from NE Florida and identified knowledge gaps for restoration planning. The second workshop focused on restoration and conservation planning using the coastal exposure map. During both workshops, we brought together a regional team with diverse experience in coastal projects to engage in communal restoration planning for the identified vulnerable sites. As an output of these workshops, we developed a table describing the most appropriate application of each elevation maintenance strategy within the context of the GTMNERR. During the workshops land managers and other stakeholders conducted a flip chart activity to identify each elevation maintenance strategy’s limitations and opportunities, which is in part reflected in Table 4. The table also includes the basic site requirements and the current knowledge gaps for each of the above-mentioned elevation maintenance strategies, as the importance of site-specific restoration and conservation approaches were shared during discussions.

For two sites of concern (N 312 and S 312)–both in the central Reserve–the model indicates that a loss of current habitats would transition more than one-quarter of this coastline to the highest exposure index level (Table 3). These sites are also partially or completely outside CBRS units and the GTMNERR (Fig. 5). We suggest the pursuit of strategies for mangrove establishment (N 312) and living shorelines or landform modification (S 312) based on the relative ease of access for restoration crews and the presence of intact marshes and newly established mangroves in the back channels. Thin-layer placement was ruled out due to the small size of local tidal channels, proximity to mangroves, and uncertainty regarding long term impacts on hydrology. Once vulnerable sites are identified by the CVI, managers can explore site-specific factors like hydrology, geomorphology, and habitat type to find an elevation maintenance strategy best suited for each site’s unique parameters, using Table 4 as a guide. With the aid of our new spatial analysis tool, regional planning in this multi-disciplinary context can build consensus for restoration and conservation action and simultaneously narrow the scope of vulnerable sites and possible restoration strategies to more manageable units for local land managers.

Field visits to sites of concern along the main channel revealed salt marsh degradation (browning marsh areas in Fig. S5B). As the tidal prism increases, water pooling inside the marsh is longer, affecting ecosystem function and coastal protection services (Shepard, Crain & Beck, 2011). The Global Surface Water dataset identifies waterlogged and potentially degraded marsh habitats (Taylor et al., 2020) that may no longer provide coastal protection services during flood events. These high-resolution datasets (30 m^2^), available from the Copernicus satellite of the European Space Agency, could further inform the marsh habitat classification and protective capacity scores utilized in this study. With a baseline established for marshes and mangroves, additional satellite image classification could map future habitat distribution, quality, and structure changes. A new research collaborative entitled “Roots and Rakes” is being organized by Villanova University and the University of Central Florida in partnership with the GTMNERR to investigate biogeochemical and geomorphic drivers of salt marsh degradation. This information can serve as a critical input to ecosystem-service assessments that account for land-sea linkages (Almenar et al., 2021), where habitat quality is used as a covariate to influence the amount of protection provided by biogenic habitats (Arkema et al., 2015; Verutes et al., 2017). Changes in coastal protection service provision could be reflected in the InVEST model by adjusting the protective distance and variable ranks where salt marshes of varying quality would range from rank 2 to 4 (high to lowest function, respectively).

## Conclusions

Extreme weather, sea-level rise, and coastal development pose significant risks to the people and ecosystems of NE Florida. A coastal exposure index was developed to screen sites of concern, and coupled with stakeholder discussion, helped to identify vulnerable areas where GTMNERR and partners have started to collect data. Through this process, we engaged land managers and scientists in a new collaboration to investigate management options that could maintain or increase wetland surface elevation with respect to sea level rise. Findings highlight near-term opportunities for elevation maintenance in vulnerable areas while considering the implications for biodiversity alongside development restrictions and land tenure.

To our knowledge, this research is the first to assess coastal vulnerability at the estuary scale for a dynamic intracoastal waterway of a National Estuarine Research Reserve. The approach advances the science underlying coastal resilience planning, habitat monitoring, and change detection by establishing a baseline of the coastal protection benefits in and around the Reserve. The GTMNERR has a clear management plan to sustain biodiversity that provides critical ecosystem-service co-benefits, such as wildlife viewing, research and education, and shoreline protection. Our findings provide spatially explicit recommendations for habitat monitoring and priority conservation areas where mangroves, salt marshes, and oyster beds are most likely to reduce risk to wetland communities of the region.

## Supplemental Information

10.7717/peerj.16738/supp-1 Supplemental Information 1 Inputs, sources, and how these data were used by the InVEST coastal vulnerability model.

10.7717/peerj.16738/supp-2 Supplemental Information 2 Scatterplot of linear regression between observed elevation and modeled elevation (LiDAR DEM).

10.7717/peerj.16738/supp-3 Supplemental Information 3 Wind rose and statistics of climatic forcing conditions that served as input to the wind exposure variable rank.

10.7717/peerj.16738/supp-4 Supplemental Information 4 Boat wake information (dot-symbols) based on interview data collected using a participatory mapping tool;. also showing AIS vessel traffic (grey shading), boating features (star-symbols), and Voronoi polygons (grey outlines).

10.7717/peerj.16738/supp-5 Supplemental Information 5 Pairwise comparisons and correlation scores of six variables in the exposure index.

10.7717/peerj.16738/supp-6 Supplemental Information 6 Photos of the greater GTMNERR study area. (A) An example of boat wakes observed along the ICW, (B) Aerial view of tidal creek and marsh vegetation, (C) Northernmost Villanova University field site, featuring the “Big Mama” mangrove, (D) Stakeholder visit in September 2021 to a potential restoration site showing the presence of oyster rakes, which are likely correlated with areas of high boating activity.

10.7717/peerj.16738/supp-7 Supplemental Information 7 Random Forest Classification of Mangrove and Salt Marsh Habitats.

## Additional Information and Declarations

### Data Availability

The following information was supplied regarding data availability: The data are available at the Resources tab of our project’s online viewer: http://cons.scienceontheweb.net/ewe/.
