# Supplementary material for: Using vulnerability assessment to characterize coastal protection benefits provided by estuarine habitats of a dynamic intracoastal waterway
Source: PeerJ. 2024 Feb 19;12:e16738. doi: 10.7717/peerj.16738 (PMC10883153; doi:10.7717/peerj.16738)
Supplement: Supplemental Information 1 [file peerj-12-16738-s001.pdf]

Table S1:
Inputs, sources, and how these data were used by the InVEST coastal vulnerability model.

Input	Source	Spatial resolution	Use of data in the model
Land mass polyline	NOAA Environmental Sensitivity Index (ESI)	1:10,000	The model uses this shape to delineate shoreline segments at a user-specified interval.
Bathymetry and elevation	University of Florida (downsampled pre-Hurricane Matthew LiDAR from the US Geological Survey; 2016)	$50m^2$	The model determines the average elevation (height in meters) of all DEM cells on land within a 2-km search radius. The resulting distribution is classified using percentile breaks to produce relative ranks of 5 through 1.
Geomorphology	NOAA Environmental Sensitivity Index (ESI); St. Johns County Shoreline Characterization (2018)	1:10,000; 30-300m transects	This input data layer will include five geomorphology classifications of each 250-meter shoreline segment ranked from 1 to 4. See Table 2 for ranking scheme.
Natural habitats	FL Fish and Wildlife Conservation Commission-Fish and Wildlife Research Institute; FL Department of Environmental Protection; National Wetlands Inventory; UF, Department of Geological Sciences	Varies	The model computes a habitat rank from 1 to 5 based on the presence or absence of habitats along each shoreline segment. See Table 2 for ranking scheme and Eq. 3 for the formula.

Input	Source	Spatial resolution	Use of data in the model
Wind exposure (local waves only)	OpenWeatherMap.org (1979-2019); NOAA's National Data Buoy Center (1989-2019)	Four weather buoys	The model computes relative wave exposure for each coastline segment by allocating the closest time series data of wind speeds and associated direction, above the 90th percentile value, and fetch distance.
Boat wakes	Automatic Identification System (AIS) vessel data from NOAA Marine Cadastre (2017-2018); Stakeholder interviews and participatory mapping (2020-2021)	Varies	AIS data for the main channel were merged with interview data focusing on the back channels and creek to characterize boat wake energy from 1 to 5 for the entire study area. See Table 2 for ranking scheme
Surge potential	The Sea, Lake and Overland Surges from Hurricanes (SLOSH v2) model from the National Weather Service (2018)	$30m^2$	Surge potential was estimated using SLOSH model outputs for a category 3 storm. The resulting distribution is classified using percentile breaks (33/66) to produce relative ranks of 2 through 4 respectively.
