# Supplementary material for: Using vulnerability assessment to characterize coastal protection benefits provided by estuarine habitats of a dynamic intracoastal waterway
Source: PeerJ. 2024 Feb 19;12:e16738. doi: 10.7717/peerj.16738 (PMC10883153; doi:10.7717/peerj.16738)
Supplement: Supplemental Information 2 [file peerj-12-16738-s002.pdf]

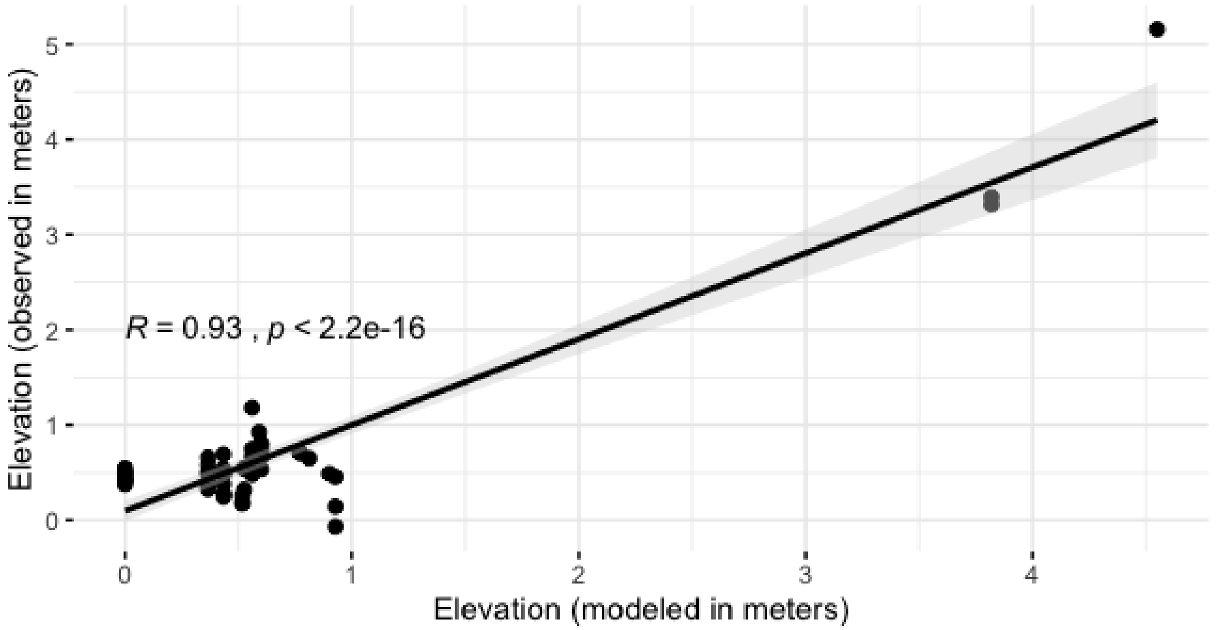

Figure S1:
Scatterplot of linear regression between observed elevation and modeled elevation (LiDAR DEM).
