# Supplementary material for: Using vulnerability assessment to characterize coastal protection benefits provided by estuarine habitats of a dynamic intracoastal waterway
Source: PeerJ. 2024 Feb 19;12:e16738. doi: 10.7717/peerj.16738 (PMC10883153; doi:10.7717/peerj.16738)
Supplement: Supplemental Information 3 [file peerj-12-16738-s003.pdf]

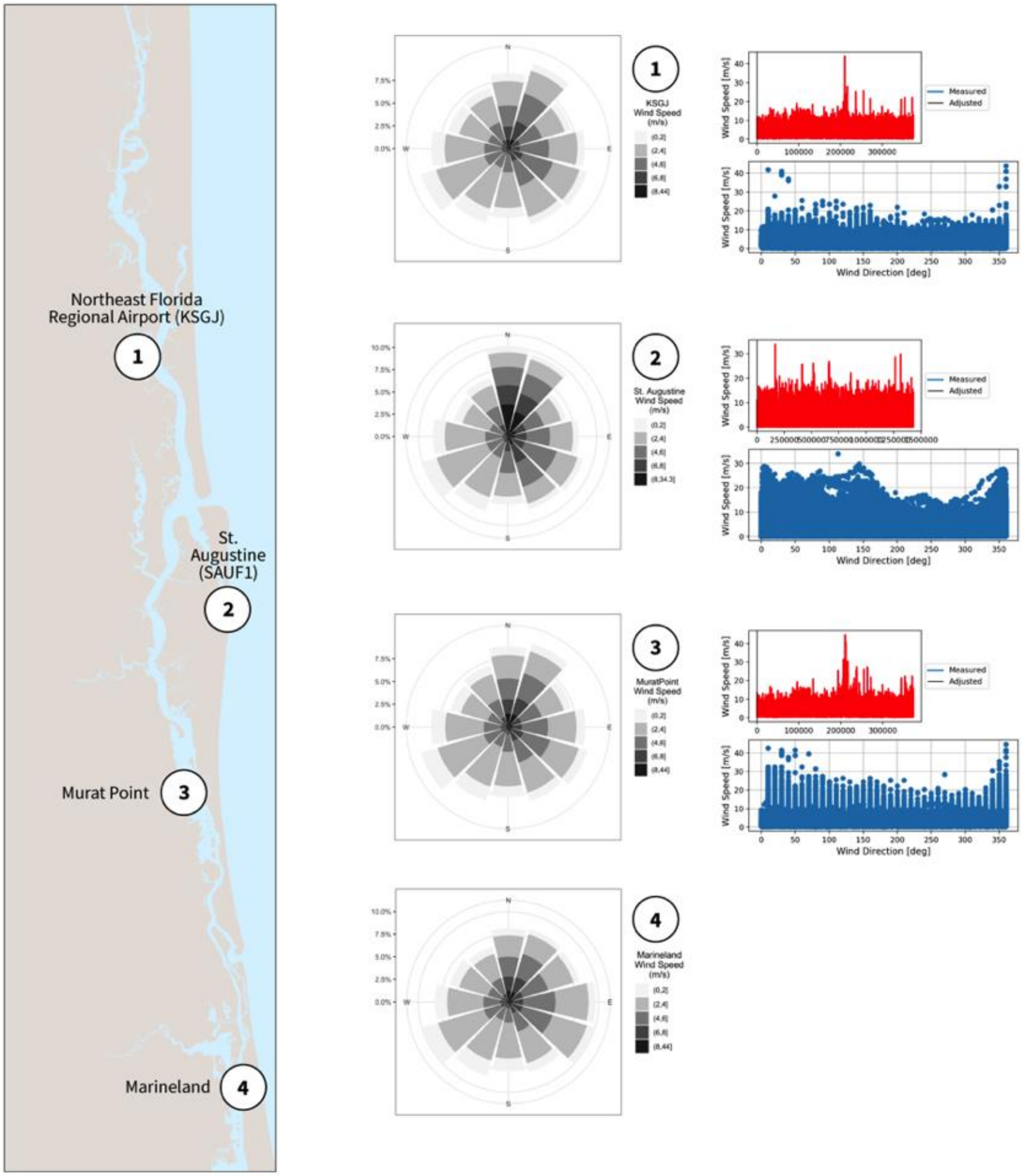

Figure S2:
Wind rose and statistics of climatic forcing conditions that served as input to the wind exposure variable rank.
