# Supplementary material for: Using vulnerability assessment to characterize coastal protection benefits provided by estuarine habitats of a dynamic intracoastal waterway
Source: PeerJ. 2024 Feb 19;12:e16738. doi: 10.7717/peerj.16738 (PMC10883153; doi:10.7717/peerj.16738)
Supplement: Supplemental Information 4 — also showing AIS vessel traffic (grey shading), boating features (star-symbols), and Voronoi polygons (grey outlines). [file peerj-12-16738-s004.pdf]

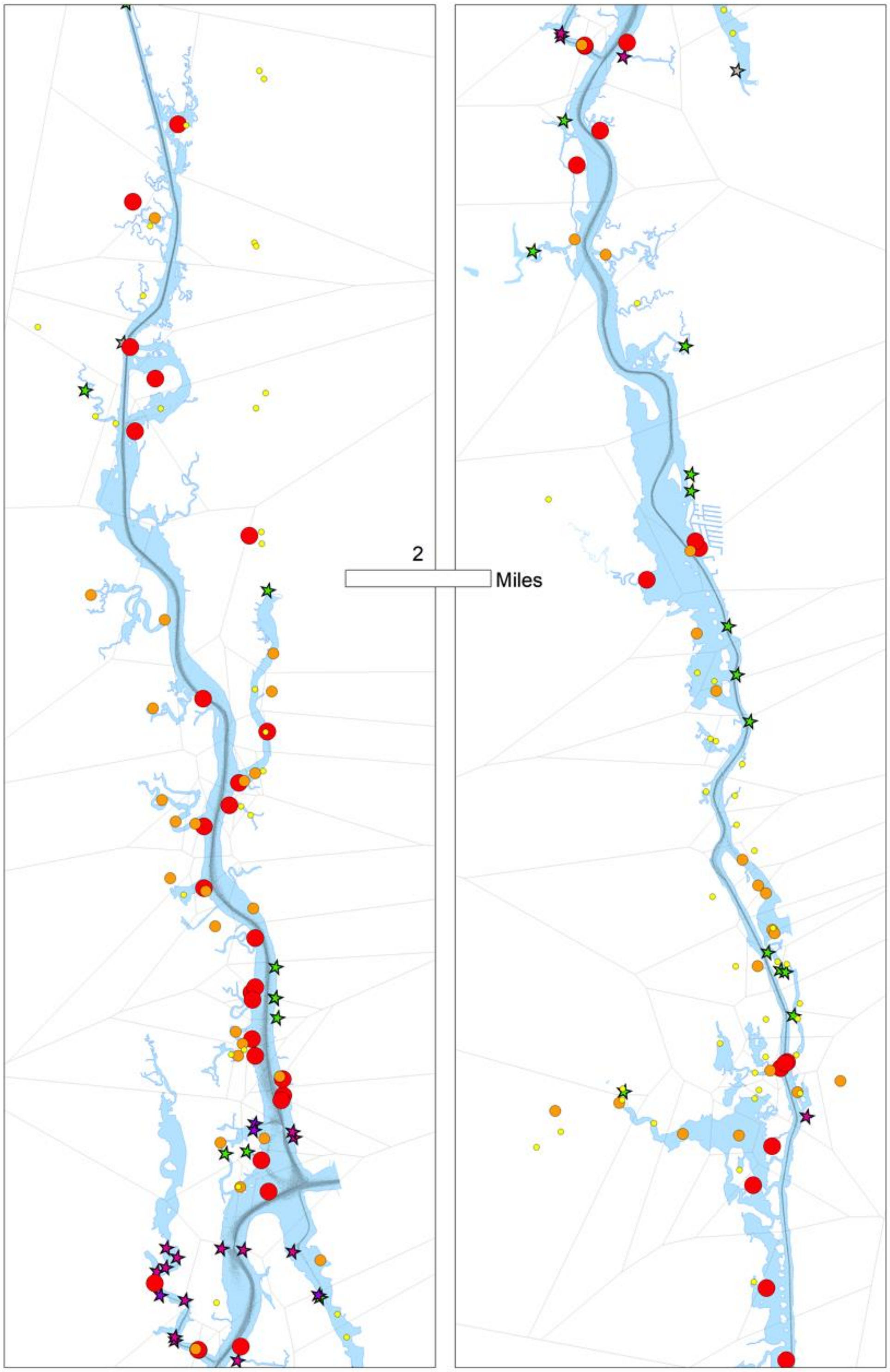

Figure S3:
Boat wake information (dot-symbols) based on interview data collected using a participatory mapping tool; also showing AIS vessel traffic (grey shading), boating features (star-symbols), and Voronoi polygons (grey outlines).
