# Supplementary figures and images for: Using vulnerability assessment to characterize coastal protection benefits provided by estuarine habitats of a dynamic intracoastal waterway
Source: PeerJ. 2024 Feb 19;12:e16738. doi: 10.7717/peerj.16738 (PMC10883153; doi:10.7717/peerj.16738)

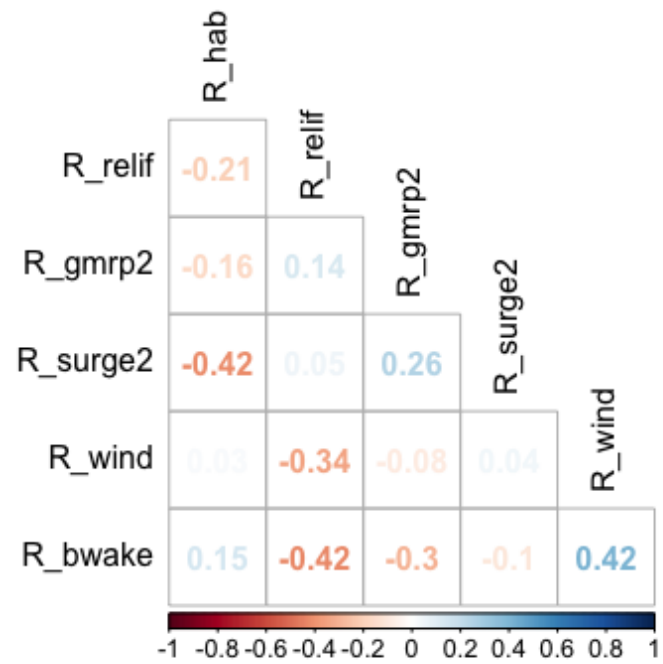

**Figure S4:**  
**Pairwise comparisons and correlation scores of six variables in the exposure index.**

Supplement: Supplemental Information 5 [file peerj-12-16738-s005.pdf]
