# Supplementary material for: Using vulnerability assessment to characterize coastal protection benefits provided by estuarine habitats of a dynamic intracoastal waterway
Source: PeerJ. 2024 Feb 19;12:e16738. doi: 10.7717/peerj.16738 (PMC10883153; doi:10.7717/peerj.16738)
Supplement: Supplemental Information 6 — (A) An example of boat wakes observed along the ICW, (B) Aerial view of tidal creek and marsh vegetation, (C) Northernmost Villanova University field site, featuring the “Big Mama” mangrove, (D) Stakeholder visit in September 2021 to a potential restoration site showing the presence of oyster rakes, which are likely correlated with areas of high boating activity. [file peerj-12-16738-s006.pdf]

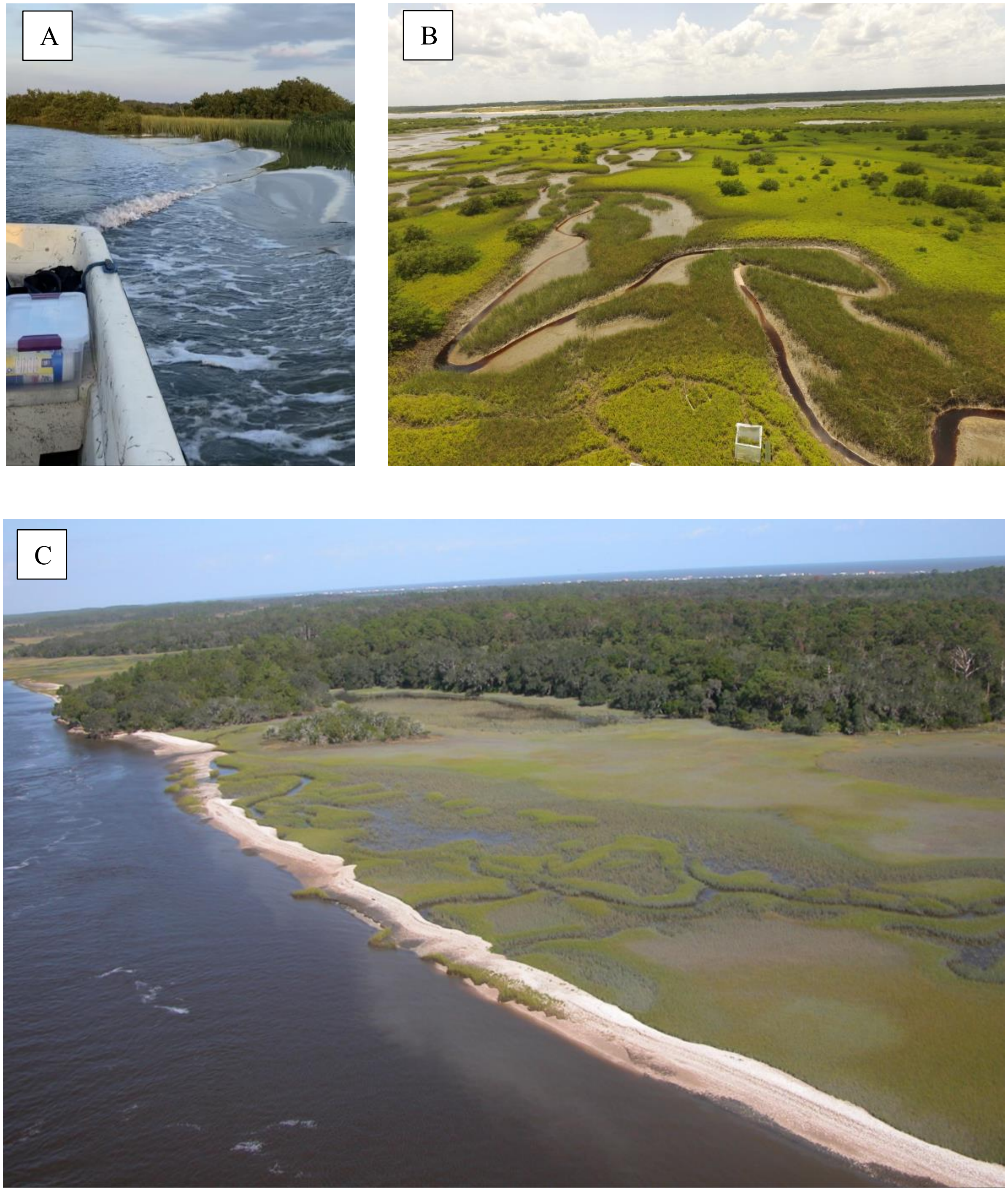
A
B
C

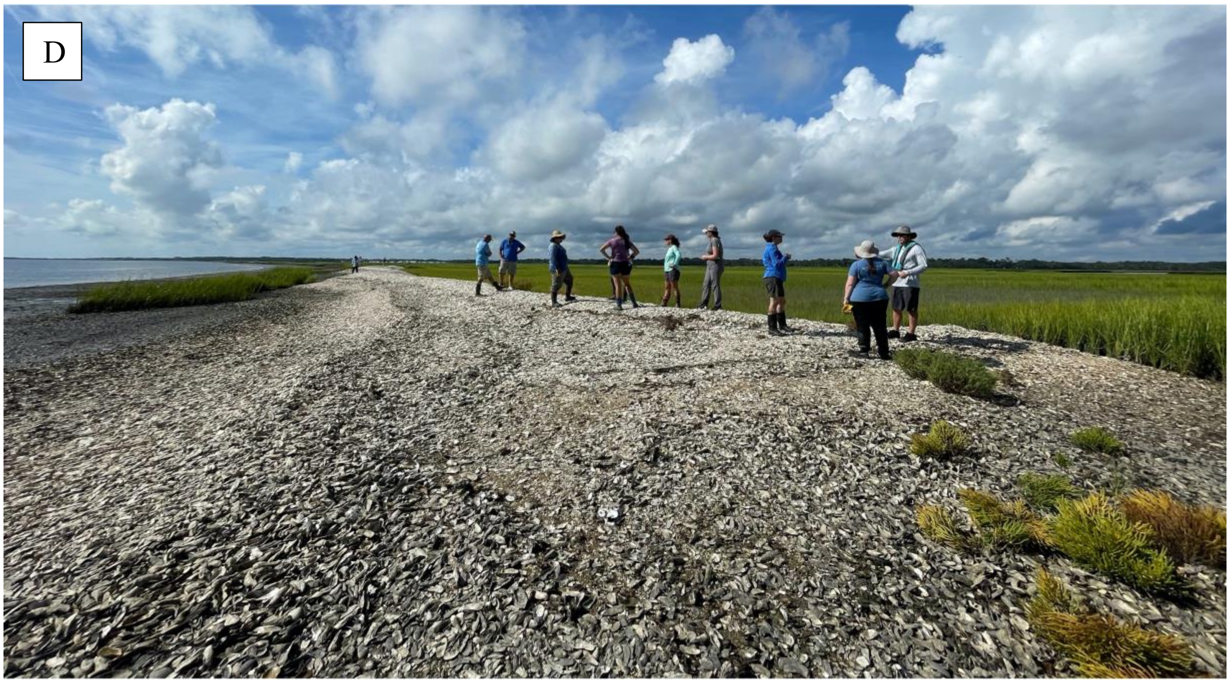

Figure S5:
Photos of the greater GTMNERR study area.
(A) An example of boat wakes observed along the ICW, (B) Aerial view of tidal creek and marsh vegetation, (C) Northernmost Villanova University field site, featuring the “Big Mama” mangrove, (D) Stakeholder visit in September 2021 to a potential restoration site showing the presence of oyster rakes, which are likely correlated with areas of high boating activity.
