# Supplementary material for: Using vulnerability assessment to characterize coastal protection benefits provided by estuarine habitats of a dynamic intracoastal waterway
Source: PeerJ. 2024 Feb 19;12:e16738. doi: 10.7717/peerj.16738 (PMC10883153; doi:10.7717/peerj.16738)
Supplement: Supplemental Information 7 [file peerj-12-16738-s007.pdf]

Supplemental Data S1:
Random Forest Classification of Mangrove and Salt Marsh Habitats

Imagery preprocessing
We created updated maps of mangrove and salt marsh habitats in GTMNERR based on imagery from the National Agriculture Imagery Program (NAIP). NAIP provides high resolution (1-m) multispectral (4-band R, G, B, NIR) imagery. We selected NAIP images taken from October to November 2017 over the study area.

NAIP images were mosaicked together using the mean of overlapping pixel values. To limit our classification analysis to relevant coastal habitats, we used the Florida Statewide Land Use Land Cover dataset to identify saltwater marshes, mangrove swamps, streams and waterways, and non-vegetated wetland habitats (FDEP, 2017). These areas were then buffered by 10 meters to include areas where mangroves may occur as fringe in other classes. The NAIP mosaic was then masked to the relevant coastal habitats. Our analysis was also limited to the GTMNERR and subset into North, Central, and South regions, each ~20 km long to help with subsetting the training data and classification analysis.

Training data
Training data were generated within areas in the FL Statewide LULC layer designated as saltwater marshes, mangrove swamps, streams and waterways, sand, and non-vegetated wetland habitats (mudflat). Points were randomly generated within each habitat class, and training samples per class were determined by the proportion of that habitat within GTMNERR. Because mangroves were underrepresented in the statewide LULC layer, the training samples for this class were increased. Training points were buffered by 10 meters to create the final training layer.

Classes of land cover (statewide LU layer) and proportion within GTMNERR AOI (247.4 km^2)

Cover class – Training data	Proportion	Training Samples per GTM Region: South	Central	North	Total Training Samples	Total Validation Samples
Mangrove	1.3 %	56	35	1	92	30
Marsh	21.5 %	113	105	190	408	135
Non-Vegetated Wetlands (Mudflats)	2.1 %	15	14	7	36	12
Sand	0.11 %	5	10	9	24	8
Water	24.6 %	84	122	169	375	125
Total					935	310

Image classification using random forest
We classified the processed NAIP mosaic into five classes (Mangrove, marsh, water, non-vegetated wetlands, and sand) using the random forest algorithm in R ("randomForest" v4.7-1; Brieman, 2001). Input data for random forest model covariates included NAIP bands (R, G, B, NIR), the normalized difference vegetation index (NDVI), and a textural filter. The texture layer was created by calculating the mean reflectance within a 5x5m window for both green and NIR bands to capture shadows created by mangrove tree canopies (Zhang, 2001). Both Green and

NIR texture layers were then averaged into a single texture layer. A random forest statistical model was then created based on the 6 data layers derived from NAIP. Data was extracted over areas corresponding to 75% of training data polygons to create the training dataset. RF models were created separately for each GTM region (south, central, north). Out of the Bag (OOB) error estimates based on our training data were 4.8% (south), 11.9 (central), and 21% (north), indicating some model prediction errors among mangrove and saltmarsh classes and saltmarsh and mudflats (central). The random forest model for each region was then used to predict the land cover class of each pixel over each region of GTM using data from the 6 data layers derived from NAIP. Output classification maps were cleaned using a sieve filter to remove classified areas less than 30 m. Marinas, docks, and bridges were manually reclassed to a new "Man-made" class, and boats and boat wakes were manually reclassed to "water".

Model validation

We used the remaining 25% of training data samples to test for the random forest model accuracy in predicting the coastal habitat classes. Overall accuracy was 91.3% based on the expected versus modeled habitat classes.

Model accuracy

	Observed - Random Forest Classification results					
Expected – Validation set	Mangrove	Mudflat	Saltmarsh	Sand	Water	Total
Mangrove	26		4			30
Mudflat		9	2	1		12
Saltmarsh		7	123		5	135
Sand				8		8
Water		6	2		117	125
Total	26	22	131	9	122	310

References

Breiman, L. 2001. Random Forests. *Machine Learning* 45: 5–32.

Florida Department of Environmental Protection (FDEP). 2017. Statewide Land Use Land Cover. FDEP Geospatial Open Data Administrator. Available from: https://geodata.dep.state.fl.us/datasets/FDEP::statewide-land-use-land-cover/about

Zhang, Y. 2001. Texture-Integrated Classification of Urban Treed Areas in High-Resolution Color-Infrared Imagery. *Photogrammetric Engineering & Remote* Sensing 67: 1359 – 1365.
